# Loss of the Yeast SR Protein Npl3 Alters Gene Expression Due to Transcription Readthrough

**DOI:** 10.1371/journal.pgen.1005735

**Published:** 2015-12-22

**Authors:** Rebecca K. Holmes, Alex C. Tuck, Chenchen Zhu, Hywel R. Dunn-Davies, Grzegorz Kudla, Sandra Clauder-Munster, Sander Granneman, Lars M. Steinmetz, Christine Guthrie, David Tollervey

**Affiliations:** 1 Wellcome Trust Centre for Cell Biology, University of Edinburgh, Edinburgh, Scotland, United Kingdom; 2 FMI Basel, Basel, Switzerland; 3 European Molecular Biology Laboratory, European Bioinformatics Institute (EMBL-EBI), Wellcome Trust Genome Campus, Hinxton, Cambridge, United Kingdom; 4 EMBL, Heidelberg, Germany; 5 The Institute of Genetics and Molecular Medicine, University of Edinburgh, Western General Hospital, Edinburgh, Scotland, United Kingdom; 6 SynthSys, University of Edinburgh, Edinburgh, Scotland, United Kingdom; 7 Department of Biochemistry and Biophysics, University of California, San Francisco, San Francisco, California, United States of America; Tufts University, UNITED STATES

## Abstract

Yeast Npl3 is a highly abundant, nuclear-cytoplasmic shuttling, RNA-binding protein, related to metazoan SR proteins. Reported functions of Npl3 include transcription elongation, splicing and RNA 3’ end processing. We used UV crosslinking and analysis of cDNA (CRAC) to map precise RNA binding sites, and strand-specific tiling arrays to look at the effects of loss of Npl3 on all transcripts across the genome. We found that Npl3 binds diverse RNA species, both coding and non-coding, at sites indicative of roles in both early pre-mRNA processing and 3’ end formation. Tiling arrays and RNAPII mapping data revealed 3’ extended RNAPII-transcribed RNAs in the absence of Npl3, suggesting that defects in pre-mRNA packaging events result in termination readthrough. Transcription readthrough was widespread and frequently resulted in down-regulation of neighboring genes. We conclude that the absence of Npl3 results in widespread 3' extension of transcripts with pervasive effects on gene expression.

## Author Summary

Npl3 is a yeast mRNA binding protein with many reported functions in RNA processing. We wanted to identify direct targets and therefore combined analyses of the transcriptome-wide effects of the loss of Npl3 on gene expression with UV crosslinking and bioinformatics to identify RNA-binding sites for Npl3. We found that Npl3 binds diverse sites on large numbers of transcripts, and that the loss of Npl3 results in transcriptional readthrough on many genes. One effect of this transcription readthrough is that the expression of numerous flanking genes is strongly down regulated. This underlines the importance of faithful termination for the correct regulation of gene expression. The effects of the loss of Npl3 are seen on both mRNAs and non-protein coding RNAs. These have distinct but overlapping termination mechanisms, with both classes requiring Npl3 for correct RNA packaging.

## Introduction

Budding yeast Npl3 comprises two RNA binding domains (RBDs) and a C-terminal domain that is rich is Arg, Gly, Ser and Tyr residues. This structure shows similarities to the SR (Ser-Arg rich) class of metazoan pre-mRNA binding proteins [1,2]. Genetic and biochemical analyses have implicated Npl3 in many processes, including pre-mRNA splicing, polyadenylation, mRNA export and cytoplasmic translation [3–7], as well as R-loop prevention and chromatin modification [6,8].

Transcription termination of RNA polymerase II (RNAPII) occurs by polyadenylation-dependent and polyadenylation-independent pathways, correlated with whether the transcript is coding or non-coding (reviewed in [9,10]). Termination of mRNAs, requires two complexes termed cleavage and polyadenylation factor (CPF) and cleavage factor (CF). Together, the CPF and CF complexes facilitate cleavage of the nascent RNA strand and removal of the elongating polymerase, resulting in a polyadenylated RNA product. Two mechanisms have been reported for these processes, which are likely to occur in combination. In the ‘torpedo’ pathway, the nascent RNA molecule is cleaved at the polyA site and the released 3’ fragment of the transcript still bound by RNAPII is degraded by the 5’-3’ exonuclease Rat1. This is proposed to then destabilize the polymerase complex. A second “allosteric” mechanism leads to the elongating polymerase being disengaged from the nascent transcript downstream of the polyA site due to, poorly understood, conformational changes concomitant with assembly of the CPF-CF complex. Notably, analyses on reporter constructs indicated that Npl3 can act as an anti-terminator, by antagonizing cleavage factor 1 (CF1) binding and thus restricting the use of cryptic poly(A) sites [4,7,11].

In addition to mRNAs, RNAPII also transcribes several classes of non-protein coding RNAs (ncRNAs) and the majority of these terminate by polyadenylation-independent pathways. These ncRNAs include the small nucleolar RNAs (snoRNAs), 73 of which function in yeast ribosome synthesis, four small nuclear RNAs (snRNAs) that form the core of the pre-mRNA spliceosome, as well as diverse long ncRNAs (lncRNAs) such as the cryptic unstable trancripts (CUTs). The snoRNAs are processed from pre-snoRNAs that can be independently transcribed, cleaved from polycistronic transcripts, or excised from pre-mRNA introns. Independently transcribed snoRNAs, snRNAs and CUTs are all thought to predominately terminate via a pathway that requires RNA-binding by Nrd1-Nab3 complex and the Sen1 helicase (together termed the NNS complex) [12–19]. Termination of snoRNAs and CUTs by the NNS complex is associated with recruitment of the TRAMP and exosome complexes to the nascent RNA [14,20–22]. The TRAMP complex tags RNAs by the addition of a short 3' oligo(A) tail, and directs target RNAs to the nuclear exosome for degradation [23–26]. This can result in either complete degradation of the RNA, in the case of CUTs, or the processing of long precursor snoRNAs to the shorter, mature form [27]. However, some snoRNAs can also be terminated by mRNA 3’ cleavage factors, with [20,28] or without [29] subsequent polyadenylation. In addition, surveillance factors can influence termination, since loss of exosome activity leads to defects in NNS termination [30–33]. Moreover, gene-length correlates with the termination pathway used, probably via changes in the phosphorylation state of RNAPII [34,35] and/or histone H3, lysine 4 trimethylation [36], both of which can promote NSS termination. Prior data indicate that a proportion of RNAPII transcription events terminate early on protein-coding genes [37–40]. These promoter proximal ncRNAs or “sCUTs” [39] are oligoadenylated, presumably by the TRAMP complex [37], and targeted for turnover by the nuclear surveillance machinery.

To better understand the in vivo functions of Npl3, we determined its RNA binding profile, and identified changes in RNA abundance and RNAPII association when the *NPL3* gene is deleted. The absence of Npl3 resulted in transcriptional termination defects at diverse RNAs, with readthrough observed on large subsets of both mRNAs and ncRNAs. These termination defects appear to cause widespread changes in gene expression, both through inappropriate termination and through transcriptional interference at neighboring genes.

## Results

### Distribution of Npl3 across the transcriptome

**Fig 1:**
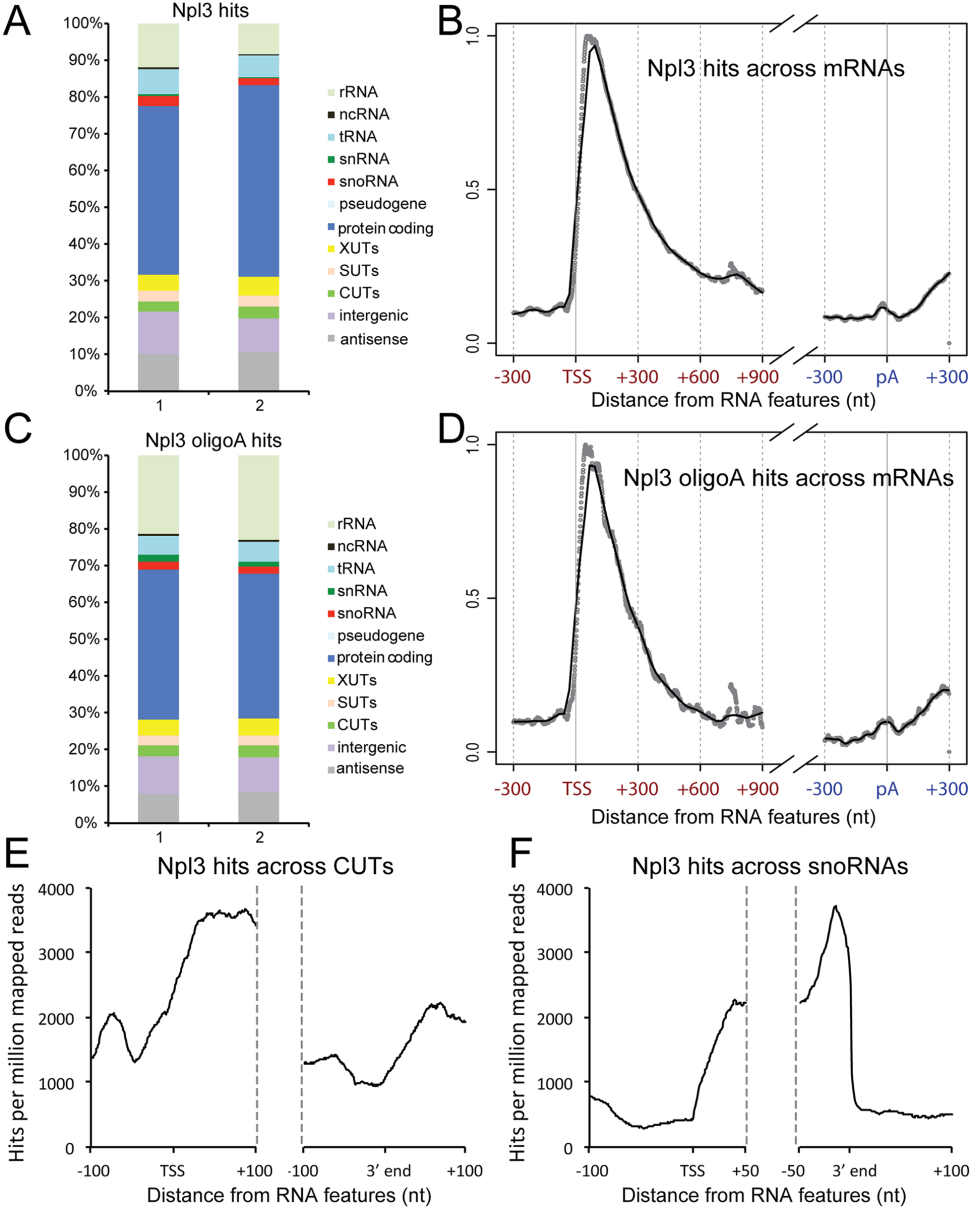
Distribution of Npl3 across RNAs is suggestive of a role in termination and surveillance. **A:** Comparison of Npl3 binding across different RNA classes in two replicate CRAC experiments. Hits were assigned to RNA classes according to gene annotations from Ensembl (EF4.74) supplemented with additional features including ncRNAs, UTRs and antisense transcripts, as previously described [37]. Antisense, intergenic and ncRNA RNA classes are defined as follows. Antisense: a hit will be assigned as 'antisense' if it maps to regions of published antisense transcription [44,45] or to the opposite strand of a known feature. Intergenic: these are hits that map to any region of the genome where no other feature has been annotated. NcRNA: this category comprises 15 ncRNAs that don't fall into other categories, including scR1, SRG1 and TLC1. **B:** Distribution of Npl3 binding over mRNAs. Average distribution of Npl3 around the 5’ and 3’ ends of mRNAs. Transcripts are aligned at the transcription start sites (TSS) and polyA (pA) site. Grey dots depict precise number of hits at particular nucleotide positions and the dark green shows a line of best fit. Hits are normalized to a total of 1 across all mRNAs. **C:** Npl3 association with oligoadenylated mRNA fragments. As for panel A, but with data filtered for the presence of non-templated oligo(A) sequences (A2 or greater) recovered on target RNAs. **D**: Distribution of Npl3 binding over mRNAs. As for 1(B), but including reads containing oligoadenylated sequences only. **E**: Metagene analysis of Npl3 binding across CUTs. Distribution of Npl3 at all CUTs aligned by the TSS and 3' ends, with 100 nt flanks extending 100 nt into the 5' and 3' ends of transcripts. Only CUTs >150 nt in length were included in the analysis. **F**: Metagene analysis of Npl3 binding across snoRNAs. Distribution of Npl3 at all snoRNAs aligned by the TSS and 3' ends, with 100 nt flanks extending 50 nt into the 5' and 3' ends of transcripts.

To identify direct RNA targets of Npl3 binding, we performed *in vivo* UV cross-linking and analysis of cDNAs (CRAC) [41]. The endogenous *NPL3* gene was tagged with an N-terminal ProteinA-TEV-His_6_ (PTH) tag, retaining the intact, endogenous *NPL3* promoter. This construct supported wild-type growth as the sole source of Npl3 (S1A Fig), indicating that the fusion protein is functional. Yeast cells were UV irradiated while actively growing and PTH-Npl3 was isolated, Npl3-bound RNA fragments were purified, converted to a cDNA library and sequenced by next generation sequencing (all sequence data are available from GEO under accession number GSE70191). S1B Fig shows expression of the tagged protein and an autoradiogram of labeled, associated RNAs. Npl3 binding sites were most frequent on mRNAs, consistent with previous studies [40,42,43], but were also identified on several classes of ncRNA, including rRNAs, tRNAs, snRNAs and snoRNAs, as well as lncRNAs, including CUTs, stable unannotated transcripts (SUTs) and other unannotated transcripts apparently derived from intergenic regions or antisense transcription. The distribution of Npl3 across RNA classes is shown for two independent CRAC experiments in Fig 1A. In both datasets, Npl3 binding was predominately on RNAPII transcripts.

The distribution of Npl3 along transcripts showed distinct patterns for different classes of RNA. On mRNAs, Npl3 binding was highest in the 5’ end region (Fig 1B), consistent with other recent RNA-crosslinking data [40]. A previous ChIP analysis, in which Npl3 is crosslinked to chromatin, found Npl3 enriched at 3' ends [6]. This apparent discrepancy may reflect differences in Npl3 binding at the 5' and 3' ends of genes, with direct RNA binding occurring predominantly at the 5' end, and stronger association with the transcription and processing complexes at the 3' end. Similar 5’ enrichment was reported for nuclear surveillance factors including Nrd1, Nab3 and Mtr4 as well as for RNAPII, and has been proposed to reflect a substantial level of premature transcription termination [37,38,40]. As described above, these promoter proximal lncRNAs are oligoadenylated by the TRAMP complex, and we therefore mapped the association of Npl3 with RNAs carrying non-encoded oligo(A) tails [37]. Among RNA fragments recovered in association with Npl3, 24–28% carried oligo(A) tails, depending on the individual CRAC experiment, indicating that Npl3 frequently binds across the junction between truncated mRNAs and oligoA tails. Note that the total fraction of Npl3 target RNAs that are oligoadenylated is likely to be higher, as only a small region of each transcript is sequenced. In contrast, only 4–4.7% of RNAs bound by RNAPII were oligoadenylated (see below). Fig 1C shows the distribution of Npl3 bound hits containing oligo(A) tails across different RNA classes for two independent CRAC experiments. The distribution of oligo(A) tails in Npl3 target sequences was similar to the overall distribution of hits on mRNAs (Fig 1D). This indicated that Npl3 is frequently bound to degradation substrates or intermediates, including prematurely terminated mRNAs, and suggests that it may function with surveillance factors to mediate early transcription termination and/or RNA degradation.

Npl3 is known to be required for efficient splicing of ribosomal protein gene (RPG) pre-mRNAs [3]. Consistent with this, we found that Npl3 strongly accumulated on introns of these pre-mRNAs relative to other intron-containing pre-mRNAs (S1C Fig). On non-RPG, intron-containing pre-mRNAs, the binding of Npl3 dropped sharply at the 5’ end of the intron. The lower recovery of introns relative to mature message indicates that Npl3 remains bound to mRNAs after splicing (S1D Fig).

The distribution of Npl3 over the CUT class of lncRNAs was similar to that observed for mRNAs with strong enrichment towards the 5' end (Fig 1E), consistent with the proposal that initial cotranscriptional packaging of pre-mRNAs and lncRNAs is similar [37]. In marked contrast, Npl3 binding was enriched towards the 3' end of snoRNAs (Fig 1F), suggesting a role in transcription termination and/or 3' end processing of these ncRNAs. Overall, our RNA binding site data suggest that Npl3 is involved in surveillance and/or transcription termination of both mRNAs and ncRNAs.

Motif analysis did not identify a specific Npl3 binding site. We note, however, that the four most overrepresented 4-nucleotide motifs each contain a U-G sequence (S1E Fig). Npl3, and particularly RRM2, was reported to show strong *in vitro* binding to U+G rich sequences including U-G dinucleotides [46].

### The absence of Npl3 results in expression changes for both coding and noncoding RNAs

To identify functional targets of Npl3, we assessed the transcriptome-wide effects of the loss of the protein on steady-state RNA levels, using strand-specific tiling arrays. Npl3 was reported to be highly abundant (78,700 copies per cell) [47] and has many different targets, which might show differential binding to residual Npl3 following depletion or relocation. We therefore analyzed the effects of deletion of the *NPL3* gene. Tiling array analyses and RNAPII crosslinking were determined using two independent strains in which *NPL3* was deleted immediately prior to the commencement of the experiments. Wild-type (WT) and *npl3Δ* strains were grown to logarithmic phase, RNA was extracted and reverse transcribed to make cDNA, which was then hybridized to tiling arrays. Normalized probe intensity data for all detected transcripts can be found in S1 Table.

**Fig 2:**
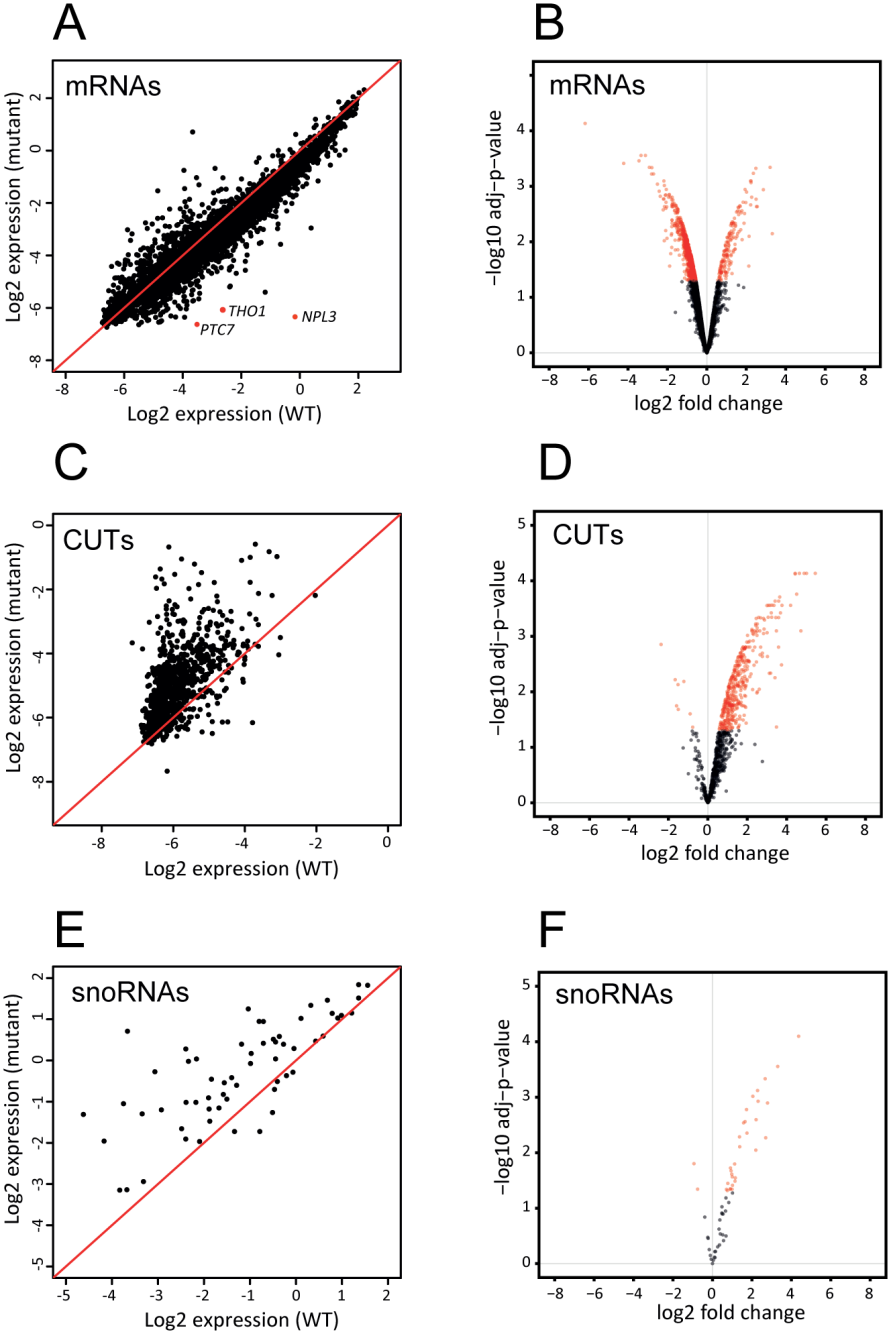
Altered RNA levels in strains lacking Npl3. Tiling microarray data were compared to assess relative RNA levels in the WT and *npl3Δ* strains for selected RNA classes; protein coding genes (A-B), CUT lncRNAs (C-D) and snoRNAs (E-F). The scatter plots (Fig 2A, 2C and 2E) display the total signal for each gene in the class for which the microarray signal was sufficiently high for quantification. Intensities are plotted on a log2 scale and the red line indicates equal intensity in the WT and *npl3*Δ mutant strains. The 3 most under-represented genes in the mutant strain–*THO1*, *PTC7* and *NPL3* –are highlighted in red. The volcano plots (Fig 2B, 2D and 2F) display differentially expressed RNAs in *npl3*Δ, with fold change plotted against p-value. The y-axis is the negative log10 p-value, adjusted to false discovery rate (FDR). The x-axis is log2 fold-change. The points in red are those that with significantly differential expression in the mutant (adj p-value <0.05). These include genes with both reduced expression (negative log2 fold change) and increased expression (positive log2 fold change).

Total RNA was extracted from WT and *npl3*Δ yeast strains, and equal amounts of cDNA were hybridized to strand-specific tiling arrays. Differential expression analysis identified 1391 mRNAs with significantly altered expression (adjusted p-value <0.05), of which 1229 were decreased and 162 were increased (Fig 2A and 2B). S2 Table shows differential expression analysis for all mRNAs, snoRNAs, CUTs and SUTs. The opposite effect was observed for CUTs, with 410 showing significantly increased expression, and only 8 showing significantly decreased expression (Fig 2C and 2D and S2 Table). Increased expression was also observed for snoRNAs; 33 showed significantly altered expression, 31 of which were increased in the mutant strain (Fig 2E and 2F and S2 Table).

### Down-regulation of mRNAs in *npl3*Δ strains due to transcriptional interference

To gain an understanding of how lack of Npl3 might lead to a global decrease in mRNA abundance, we ranked all mRNAs by log2 fold change in the mutant compared to the WT strain, according to the differential expression analysis (S2 Table). We then focused our analyses on the 30 most down-regulated genes in the *npl3Δ* strain, and examined their genomic environment (Table 1). As expected, the most down-regulated gene was *NPL3*, which is absent from the genome and was discounted from the analysis. We found that 15/30 (50%) of down-regulated genes reside in a convergent orientation with an expressed protein coding gene. A previous analysis found that only 6% of all yeast genes reside in convergent orientations in which both genes are expressed [48]. The proportion of convergent mRNAs with reduced expression in *npl3*Δ strains was therefore unexpectedly high. At 11 of the 15 convergent mRNA loci (73%), the down-regulated gene is adjacent to a gene that showed clear transcription readthrough, suggesting that their expression is blocked by transcriptional interference. An additional nine down-regulated mRNAs are convergent with an ncRNA that showed transcription readthrough. A further four down-regulated mRNAs are located in tandem with an upstream gene that shows readthrough, while seven mRNAs are apparently down-regulated by both tandem and convergent readthrough. Three of the 30 most down-regulated genes do not appear to be inhibited by convergent or tandem readthrough, or by intergenic transcription. Of these, *YJR015W* is seemingly down-regulated due to transcription changes over a local chromosome domain, since both upstream tandem genes are also down-regulated, while *FMP48* and *TPO4* are down-regulated by unknown mechanisms.

**Table 1:** 

Gene	Expression level^a^	Orientation^b^ (nearest mRNA)	Convergent mRNA RT?	Convergent ncRNA RT?	Tandem RT?
NPL3	-6.17	N/A			
YRO2	-4.22	C	Y		Y
THO1	-3.44	C	Y		
HXT1	-3.34	T			Y^d^
PTC7	-3.12	C	Y		
YJR015W^c^	-2.92	C			
CYC1	-2.80	C	Y		Y
YPR172W	-2.79	C	Y		
TPO2	-2.70	C	Y		
HSP30	-2.51	C			Y
FMP48	-2.42	C			
TPO1	-2.36	T		Y^d^	Y^d^
STP4	-2.22	T			Y
UNG1	-2.20	C	Y		
PTC2	-2.18	T		Y	
ADE17	-2.18	C	Y		
ISF1	-2.16	T			Y^c^
HXT4	-2.16	T		Y	
FMP12	-2.15	T		Y	
EFM3	-2.11	T		Y	
FMP41	-2.09	T			Y^d^
GSY1	-2.09	T			Y
SIA1	-2.08	C	Y		
TPO4	-2.07	C			
SCS7	-2.06	T		Y	Y^d^
MIG3	-2.04	T		Y	
WSC4	-2.02	T		Y	
PTI1	-1.94	T		Y	
HOR2	-1.92	T			Y
IML3	-1.90	C	Y		Y^d^
POA1	-1.89	C	Y		Y^d^

^a^ Expression determined as Log2 fold change in mutant relative to WT

^b^ Orientation determined with respect to 3' end of listed gene; C = convergent, T = tandem

^c^ No RT observed at this gene but two previous genes down-regulated indicating local transcription changes

^d^ Readthrough phenotype subtle

Although mRNA expression was most frequently decreased in *npl3*Δ strains, several mRNAs were up-regulated. We examined the genomic environment for the top 30 up-regulated genes (S3 Table). Eleven of these correspond to spliced ribosomal protein genes, and increased intron signal in the *npl3*Δ strain accounts for the differential expression. A further eleven up-regulated genes showed increased readthrough from upstream mRNAs or ncRNAs, suggesting that apparent increased expression is due to readthrough signal from the neighboring gene rather than specific up-regulation. The remaining eight genes (*HSP12*, *DDR2*, *HES1*, *YDR124W*, *YML007C-A*, *ALP1*, *PUG1* and *YCL049C*) are apparently specifically up-regulated in *npl3*Δ strains.

### Expression changes at convergent gene loci

**Fig 3:**
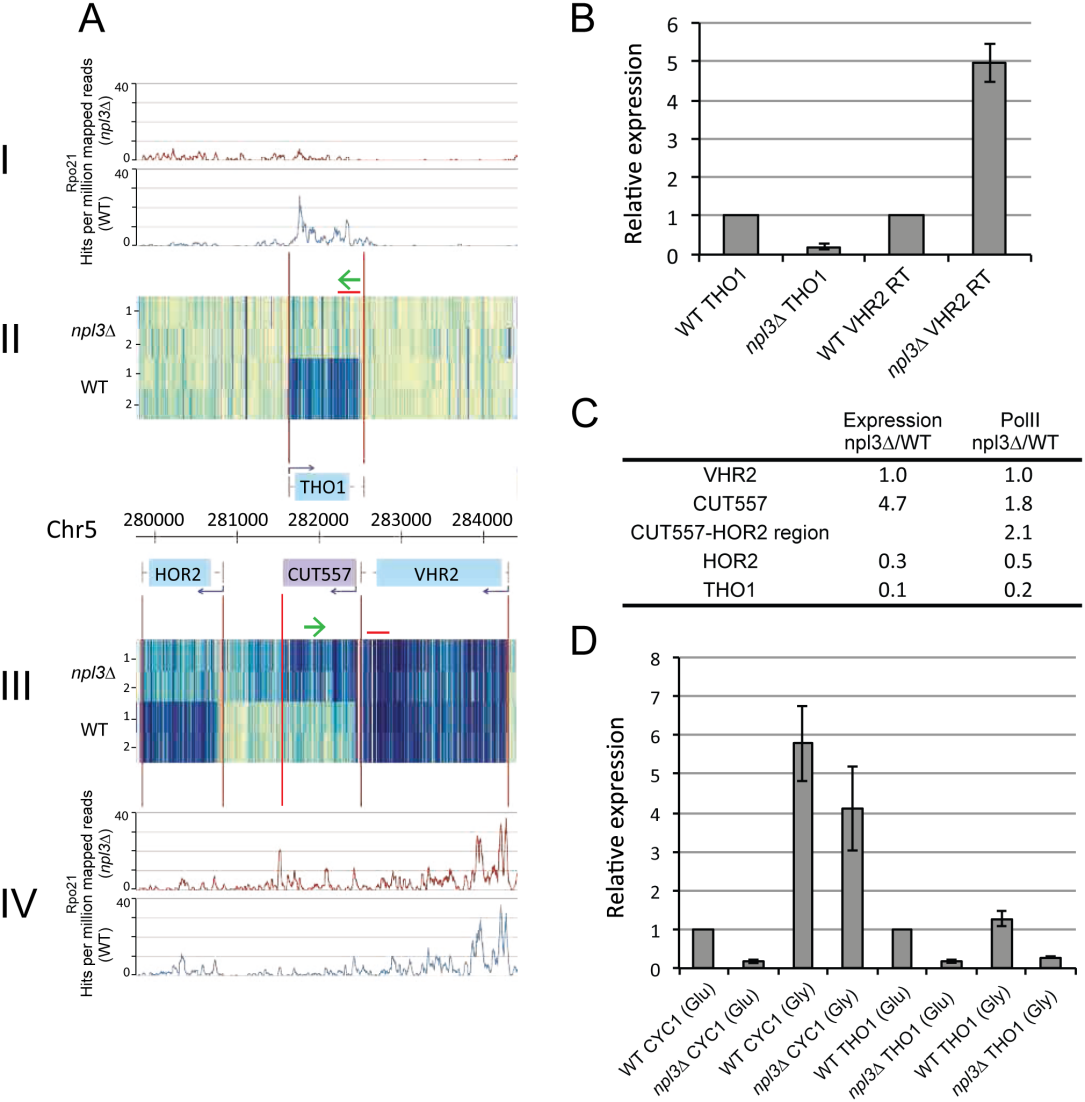
Transcriptional readthrough at the *VHR2-THO1* locus. **A:** Expression (II-III) and polymerase occupancy (I, IV) at the *VHR2-THO1* locus on chromosome V, in WT and *npl3*Δ mutant yeast. Expression was determined using strand-specific tiling arrays, and two biological replicates are shown for both yeast strains (tracks labeled 1 and 2). Expression from the Watson strand is shown above the genomic co-ordinate information, and expression from the Crick strand is shown below. Polymerase (Rpo21) occupancy on each strand is shown in blue (WT) or red (*npl3*Δ). **B:** Confirmation of transcriptional readthrough of *VHR2* (VHR2 RT) and down-regulation of *THO1* in *npl3*Δ using strand- specific reverse transcription followed by qPCR. Approximate locations of primers used for reverse-transcription are shown in 3(A) (green arrows). *VHR2* readthrough is measured by performing a reverse transcription reaction using an oligo that primes from ~500 nt downstream from the *VHR2* 3' end. qPCR primers are located towards the 3’ end of *THO1* and *VHR2*, respectively. The histogram shows changes in the *npl3Δ* mutant, compared to the WT levels (which were set to 1). **C:** Comparison of tiling array expression data and polymerase occupancy at regions across the *VHR2*-*THO1* locus, in WT and *npl3*Δ. Numbers represent change in the *npl3*Δ mutant relative to WT. **D**: Induction of *CYC1* in glycerol. Induction of *CYC1* expression in WT and *npl3*Δ yeast transferred from glucose to glycerol medium, determined by reverse-transcription followed by qPCR. *THO1* expression was measured in both conditions, as a negative control.

To investigate whether the gene expression changes observed are indeed due to transcriptional interference, we more closely analyzed two strongly down-regulated mRNAs: *THO1* and *PTC7* (Figs 3 and S2, respectively). In Fig 3A, panels II and III show tiling array expression data for two biological replicates of the *npl3Δ* strain (upper) and WT (lower) strains in a genome viewer format. Panels I and IV show corresponding data for the association of RNAPII with the nascent transcript as determined by UV-crosslinking and analysis of cDNAs (CRAC; see below). Features on the Watson strand are shown above the chromosomal nucleotide numbers and features on the Crick strand are shown below. Apparent readthrough from the *VHR2* gene is associated with strong down-regulation of *THO1*, which encodes a nuclear pre-mRNA binding protein (Fig 3A). Strand-specific reverse transcription (RT), followed by qPCR confirmed that the *VHR2* gene was indeed extended, and that the increased downstream expression was not a distinct transcription product (Fig 3B). Quantification by RT-qPCR indicated that 3’ extended *VHR2* is elevated ~5 fold, whereas *THO1* expression is reduced ~5 fold. The approximate positions of RT primers and qPCR amplicons are shown by green arrows and red lines, respectively, in Fig 3A.

Similar analysis of the *UPF2*-*PTC7* region revealed that apparent readthrough from the *UPF2* gene is associated with strongly reduced expression of *PTC7*, encoding a Type 2C serine/threonine protein phosphatase (PP2C) (S2A Fig). In this case, RT-qPCR quantification revealed ~10 fold elevated readthrough from *UPF2*, associated with ~6 fold suppression of *PTC7* expression (S2B Fig). This suggests that transcription termination defects in the *npl3Δ* strain lead to changes in expression of surrounding genes.

### RNAPII occupancy confirms mRNA termination defects in the absence of Npl3

It remained possible that changes in RNA abundance for the *npl3Δ* strain observed in tiling array and RT-qPCR data might reflect reduced pre-mRNA surveillance and degradation rather than altered transcription. To discriminate between increased readthrough and RNA stabilization, we assessed changes in RNAPII occupancy following loss of Npl3. To do this, we used CRAC to crosslink RNAPII to the nascent transcript, which provides genome-wide, strand-specific, nucleotide resolution mapping data *in vivo* in growing cells. The CRAC technique was applied using strains in which the largest subunit of RNAPII, Rpo21, carried a C-terminal, His6-TEV-Protein A (HTP) tag, as recently described (Milligan et al., submitted). Tagged Rpo21 was well expressed in WT and *npl3Δ* strains, and was shown to crosslink efficiently to RNA (S3A Fig). Total RNAPII occupancy across different classes of RNA was largely unchanged between the WT and *npl3*Δ strains (S3B Fig). However, significant differences in the location of RNAPII were observed for individual genes. In Figs 3A and S2A, blue plots show Rpo21 occupancy in WT yeast and red plots show occupancy in *npl3Δ*. The density of RNAPII was highest at the 5’ ends of most protein-coding genes, consistent with published NET-seq data that maps the transcribing polymerase by sequencing 3' ends of associated nascent transcripts [38], and with the distribution of pre-mRNA binding factors, including Npl3 ([37,40] and Fig 1). Differences in RNAPII occupancy at the two convergent loci are summarized in Figs 3C and S2C. RNAPII occupancy within the *VHR2* ORF was comparable between the two strains, and RNA accumulation was very similar in the mutant and WT strains (Fig 3A and 3C). However, in the *CUT557* region immediately downstream, RNA accumulation was increased 4.7 fold while polymerase occupancy was increased 1.8 fold in *npl3Δ*. RNAPII crosslinking in the region between *CUT557* and the downstream gene *HOR2* was also elevated by 2.1 fold in the mutant, indicating that transcriptional readthrough extends into this region. *HOR2* itself appears to be inhibited by transcriptional interference acting in tandem, as shown by decreased RNA accumulation (to 30% of WT), and polymerase occupancy (decreased to 50% of WT). The *THO1* transcript is greatly reduced in *npl3Δ* (to 10% of WT), with polymerase occupancy reduced to 20% of WT.

Analysis of expression and RNAPII occupancy over the *UPF2-PTC7* locus also confirmed *UPF2* readthrough and *PTC7* down-regulation (S2A and S2C Fig). In addition, RNAPII density was decreased over the downstream *PPE1* gene. This indicates that the transcriptional readthrough from *UPF2* also inhibits expression of this tandem, flanking gene. Down-regulation of *PPE1* can only be determined from the RNAPII occupancy data and is not evident from tiling array data as the *PPE1* signal is obscured by the *UPF2* readthrough signal. This demonstrates the difficulty in discriminating down-regulation due to readthrough in tandem. We conclude that transcriptional readthrough of multiple mRNA genes results in down-regulation of downstream convergent and tandem genes.

### Decreased abundance of polyadenylated UPF2 mRNA in *npl3*Δ

To determine whether correctly processed and polyadenylated mRNAs are also produced from genes showing transcriptional readthrough, we analyzed the 3' end of *UPF2* in WT and *npl3*Δ by cleavage with RNase H using an oligo hybridizing ~250 nt upstream of the *UPF2* annotated 3’ end. Cleavage reactions were performed with the gene-specific oligo, with and without the addition of oligo(dT) to deadenylate the cleavage product (S2D Fig). We observed substantially less mature polyadenylated *UPF2* mRNA in the mutant (lanes 1 and 2, compared to 4 and 5), but the adenylation pattern was apparently the same (lane 2 compared to 5). This indicates that cleavage and polyadenylation of *UPF2* mRNA is reduced in the *npl3Δ* strain, but the location of the residual activity is unaltered.

### Down-regulation by transcriptional readthrough does not preclude transcription regulation

The tiling array data indicate that expression of the *CYC1* gene is down-regulated in *npl3Δ* due to transcriptional readthrough from the convergent gene *UTR1* (Table 1). *CYC1* encodes cytochrome C and transcription is up-regulated on glycerol medium. WT and *npl3Δ* strains were grown in either glucose or glycerol medium and the level of *CYC1* mRNA was quantified by RT-qPCR (Fig 3D). On glucose medium *CYC1* was reduced ~5.9 fold in *npl3Δ* relative to WT, validating the findings of the tiling array. However, *CYC1* abundance was increased 4.1 fold when the *npl3Δ* strains were transferred to glycerol medium, resulting in an expression level close to WT. In contrast, the level of *THO1* was not increased by transfer of the *npl3Δ* strain to glycerol medium (Fig 3D). This demonstrates that *CYC1* expression remains subject to specific transcription regulation in the absence of Npl3.

### Npl3 is required for efficient termination of ncRNA transcription

Npl3 was crosslinked to ncRNAs (Fig 1) and the *npl3*Δ mutation altered the expression of ncRNAs including CUTs and snoRNAs (Fig 2), suggesting that the loss of Npl3 might also affect transcription termination on ncRNA genes.

**Fig 4:**
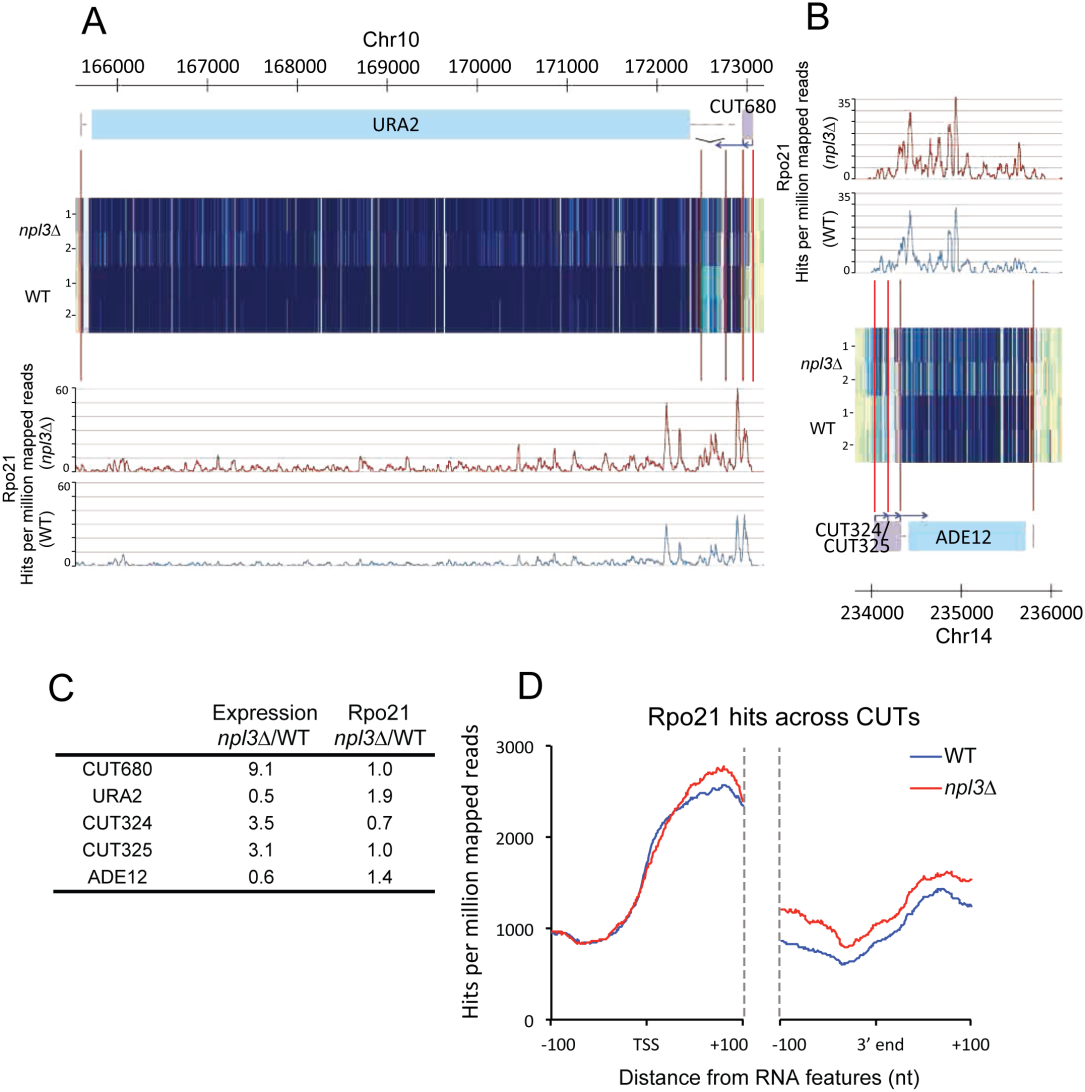
Transcription termination defects at CUTs in *npl3*Δ. **A:** Expression and polymerase occupancy at the CUT680/URA2 locus, in WT and *npl3*Δ mutant yeast. Expression is determined using strand-specific tiling arrays, and two biological replicates are shown for each yeast strain (heat map tracks labeled 1 and 2). Polymerase occupancy was determined by CRAC, and is shown for WT (blue) and *npl3*Δ (red) yeast. Note there is an intron at the 5' end of *URA2*. **B:** Expression and polymerase occupancy at the *CUT324/CUT325/ADE12* locus. Data displayed as in panel A. **C:** Comparison of tiling array expression data and polymerase occupancy at regions across the *URA2* and *ADE12* loci, in WT and *npl3*Δ. Numbers represent change in the *npl3*Δ mutant relative to WT. **D**: Metagene analysis of Rpo21 binding across CUTs. Distribution of Rpo21 at all CUTs in WT (blue) and *npl3*Δ (red) yeast, aligned by the TSS and 3' ends, with 100 nt flanks extending 100 nt into the 5' and 3' ends of transcripts. Only CUTs >150 nt were included in the analysis.

Previous work identified genes that are regulated by upstream CUTs, which inhibit transcription of the downstream mRNA, including the nucleotide biosynthesis factors *ADE12* and *URA2* [49]. In *npl3*Δ strains, CUT680 upstream of *URA2* and CUT324/325 upstream of *ADE12* were accumulated, accompanied by reduced expression of the downstream protein-coding gene (Fig 4A–4C). Metagene analyses show increased polymerase density at the 3' ends of CUTs, and immediately downstream, in *npl3*Δ compared to WT (Fig 4D). These data suggest that that Npl3 is required for normal termination of CUTs, and that without proper termination these normally unstable transcripts are not efficiently turned over by the nuclear RNA surveillance machinery.

**Fig 5:**
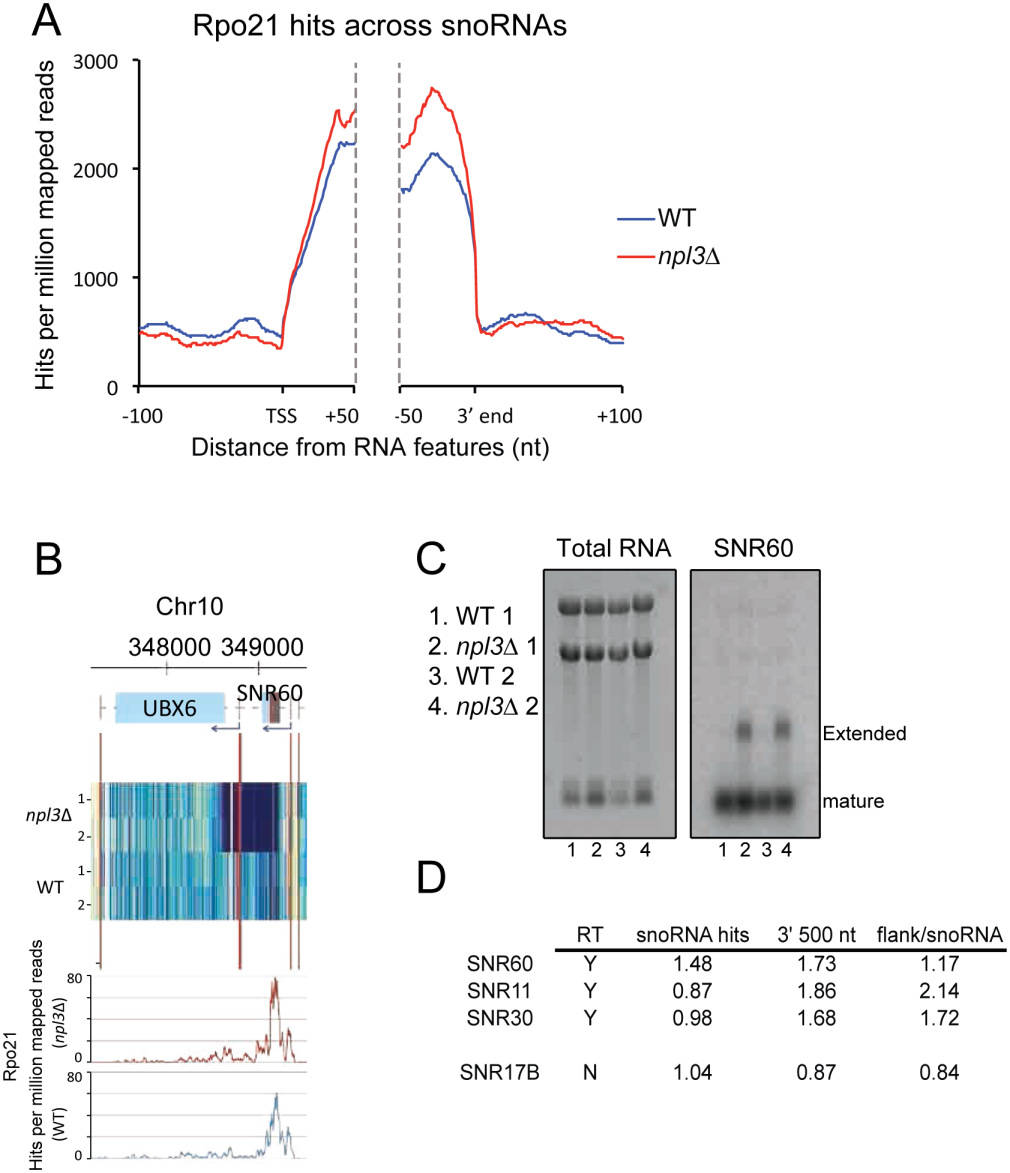
Transcription termination defects at snoRNAs in *npl3*Δ. **A**: Metagene analysis of Rpo21 binding across snoRNAs. Distribution of Rpo21 at all snoRNAs in WT (blue) and *npl3*Δ (red) yeast, aligned by the TSS and 3' ends, with 100 nt flanks extending 50 nt into the 5' and 3' ends of transcripts. **B:** Expression and polymerase occupancy at SNR60. Data displayed as in 4(A). **C:** Northern blot to detect extended SNR60 in *npl3*Δ (right panel). The left panel displays total RNA. Two biological replicates are shown for each strain, WT and *npl3*Δ. **D**: Polymerase occupancy within and flanking selected snoRNAs.

Inspection of microarray data revealed 3’ extensions for many snoRNAs in *npl3Δ* strains. All H/ACA and C/D box snoRNAs were included in the analysis and, strikingly, we observed extended 3’ ends for 46 of the 51 RNAPII transcribed, monocistronic snoRNA genes, and for all five polycistronic pre-snoRNA transcripts. One gene (*SNR13*) could not be interpreted due to missing probes (Tables 2 and S4). Another, SNR52, is the sole snoRNA transcribed by polymerase III, and is therefore terminated through a different pathway. This leaves just three RNAPII transcribed snoRNAs that do not show readthrough: U3B (*SNR17B*), *SNR63* and *SNR85*. Metagene analyses of the Rpo21 CRAC data showed increased RNAPII association towards the 3' ends of all snoRNAs in *npl3Δ* strains (Fig 5A).

**Table 2:** 

ncRNA class	snoRNA transcript class	Number showing RT
snoRNAs	All	51/57 (77 total)^a^
	Monocistronic	46/51
	Polycistronic	5/5 (17 total)^b^
	Intronic	N/A (8 total)
snRNAs	N/A	0/5

^a^ This includes all H/ACA and C/D box snoRNAs. Of the 76 snoRNAs, only 56 have the potential to show read-through, being either monocistronic or the final snoRNA in a polycistronic transcript.

^b^ 17 polycistronic snoRNAs are encoded on 5 separate transcripts.

Examples of extended snoRNAs are shown in Figs 5 and S4. The box C/D snoRNA snR60 is extended approximately 500 nt in *npl3Δ* and appears to terminate about 100 nt into the downstream *UBX6* gene (Fig 5B). The presence of extended snR60 was confirmed by northern blot (Fig 5C). S4 Fig shows extension of the box H/ACA snoRNA snR3, determined by tiling array, and RNAPII occupancy data (S4A Fig) and confirmed by RT-qPCR (S4B Fig). Comparison of expression and RNAPII occupancy at this locus is shown in S4C Fig. The snR3 transcript appears to be extended greater than 1000 nt downstream with transcription proceeding through downstream, annotated CUT genes (*CUT221/222/223*).

In some cases, extension of snoRNA genes was associated with strongly reduced expression of neighboring genes. As an example, *SNR3* readthrough correlates with reduced expression of *EFM3* (S4A–S4C Fig). Some snoRNAs appear to be extended many kilobases, apparently utilizing the termination site of the next downstream protein gene. To confirm that snoRNA 3’ extensions result from transcriptional readthrough, we calculated “readthrough scores” for three snoRNAs (*SNR11*, *SNR30* and *SNR60*) that appeared to be extended based on tiling array data, as well as *SNR17B* that did not appear to be extended. We calculated the sum of all RNAPII hits in the 500 nt 3’ flanking region, relative to the sum of all hits within the snoRNA sequence, and compared this ratio for the WT and *npl3Δ* strains. For the extended snoRNAs, Rpo21 hits in the 3’ flanking region hits were elevated 1.16 to 2.17 fold in *npl3Δ*, but reduced to 0.84 fold of the WT for *SNR17B* (Fig 5D). Overall, the magnitude of RNAPII occupancy changes downstream of snoRNAs in *npl3*Δ relative to WT is much less than changes in expression. We suggest that the extended snoRNA transcripts predominately reflect defects in RNA surveillance rather than processing/maturation, as we found the abundance of mature snoRNAs to be comparable in the *npl3*Δ mutant and WT strains (S4D Fig).

Many snoRNAs harbor a cleavage site for the endonuclease Rnt1 (RNase III) positioned downstream of the mature 3’ end (reviewed in [50]). Cotranscriptional cleavage by Rnt1 provides an entry site for 3’-exonuclease processing back to the mature 3’ end of the snoRNA, and also allows the 5’ exonuclease Rat1 to degrade the nascent transcript and terminate the transcribing polymerase [51–58]. We therefore predicted that snoRNAs possessing 3' Rnt1 cleavage sites would not exhibit readthrough in *npl3*Δ strains. Unexpectedly, however, there was no apparent correlation between readthrough transcription in the *npl3Δ* strain and the presence or absence of reported Rnt1 cleavage (S4 Table).

No extension was seen on any of the RNAPII transcribed snRNAs (U1, U2, U4 or U5) in the *npl3Δ* strain (Table 2). It had appeared that snRNAs and snoRNAs utilize related termination pathways [59] and a recent study found extended forms of both snoRNAs and snRNAs in strains lacking Rrp6 [31]. Furthermore, as for snoRNAs, Rnt1 cleavage sites flank the U1, U2, U4 and U5 genes [50]. However, despite these apparent similarities, there are clear differences in their requirement for Npl3.

### Changes in Nab3 recruitment in *npl3*Δ

Strains lacking Npl3 show transcription readthrough on protein coding genes, on which termination generally requires the cleavage and polyadenylation machinery, and on ncRNA genes that are terminated by the Nrd1-Nab3-Sen1 (NNS) complex. The NNS complex is implicated in termination of CUTs, snoRNAs and some mRNAs and physical interactions have been reported between Npl3 and the NNS components [60,61]. We therefore investigated whether this complex is properly recruited in *npl3*Δ. RNA crosslinking by Nab3 was more efficient than by Nrd1, so we focused our analyses on this protein.

To assess recruitment of the NNS complex we applied the CRAC approach to Nab3-HTP. The *npl3*Δ strain expressing tagged Nab3 grows very slowly (doubling time 6h), indicating a negative genetic interaction. However, Nab3-HTP was well expressed in *npl3Δ* and crosslinked to RNA with even greater efficiency than in the WT (S5A Fig). Crosslinking of Nab3 to different RNA classes was similar in *npl3*Δ and WT strains (S5B Fig). Nab3, like Npl3, binds strongly at the 5' ends of mRNA transcripts (S5C Fig) and showed a substantial frequency of non-templated oligo(A) tails (36% in two experiments) consistent with active surveillance in this region.

**Fig 6:**
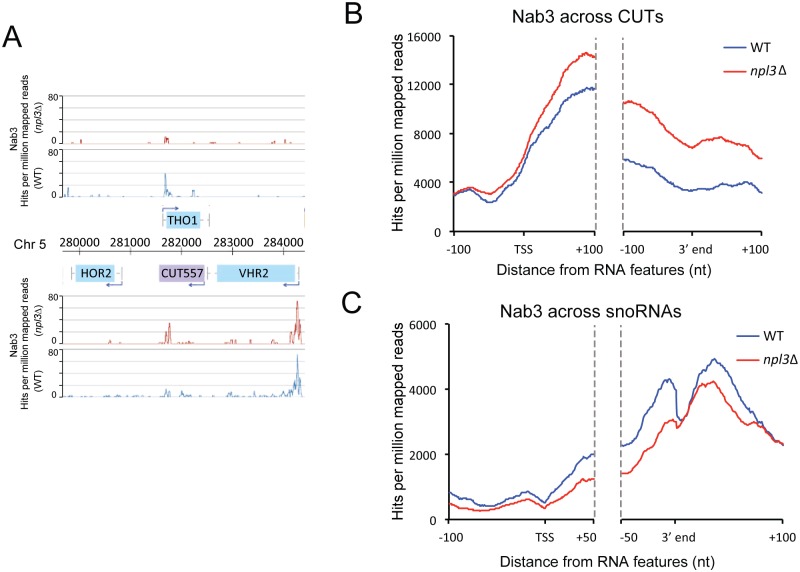
Transcriptome-wide binding of Nab3 in WT and *npl3*Δ. **A**: Nab3 binding at the *VHR2-THO1* locus, in both WT (blue) and *npl3*Δ (red) yeast. **B**: Metagene analysis of Nab3 binding across CUTs. Distribution of Nab3 at all CUTs in WT (blue) and *npl3*Δ (red) yeast, aligned by the TSS and 3' ends, with 100 nt flanks extending 100 nt into the 5' and 3' ends of transcripts. Only CUTs >150 nt were included in the analysis. **C**: Metagene analysis of Nab3 binding across snoRNAs. Distribution of Nab3 at all snoRNAs in WT (blue) and *npl3*Δ (red) yeast, aligned by the TSS and 3' ends, with 100 nt flanks extending 50 nt into the 5' and 3' ends of transcripts.

Inspection of the *VHR2-THO1* convergent gene locus (Figs 3 and 6A) revealed strong peaks of Nab3 binding at the 5’ ends of *VHR2* and *THO1*, reflecting the role of NNS in early termination on protein coding genes. In the *npl3Δ* strain the peak at the 5’ end of *VHR2* was unaltered, whereas the peak on *THO1* was lost due to transcription interference. A peak of Nab3 towards the 3’ end of *CUT557* presumably reflects the known role of NNS in CUT termination. Notably, this peak was increased when Npl3 is absent, corresponding with the increased *CUT557* expression. We conclude that the *VHR2-CUT557* readthrough transcripts are likely to be terminated by the NNS pathway rather than by the CPF-CF pathway. Nab3 binding across CUTs was strongly increased in *npl3*Δ, particularly around the 3' ends of these transcripts and at downstream sites (Fig 6B). The increased binding of CUTs by Nab3 in *npl3*Δ was greater than the increased RNAPII association we observe in the mutant strain (Fig 4D) suggesting that it reflects not only increased expression of these ncRNAs, but additional non-productive recruitment of this surveillance factor to normal degradation substrates. On snoRNAs we observe a contrasting phenotype, with reduced Nab3 binding across the length of the transcript in *npl3*Δ strains (Fig 6C). Decreased Nab3 association with snoRNAs may be related to the apparent processing defect, since the NNS complex helps promote 3’ maturation by recruitment of the exosome [27].

Overall our Nab3 binding data suggest that readthrough transcripts are targets of the NNS complex, demonstrated by increased binding of Nab3 in the extended region in *npl3*Δ compared to WT. In the mutant strain we see a shift in Nab3 binding away from processing targets (snoRNAs) onto surveillance targets (CUTs and extended mRNAs). This might explain why the *npl3*Δ/Nab3-HTP strain displays a synergistic growth defect. Efficient recruitment of Nab3 is likely to be more critical in an *npl3*Δ strain, in which many surveillance targets are produced. Mild interference with recruitment due to the tag might therefore have a negative effect on growth in the *npl3Δ* background, despite giving no clear phenotype in the WT.

Widespread termination defects result in genome-wide expression changes. We next used individual probe intensity data from the tiling arrays to calculate the level of readthrough genome-wide. Three windows were defined for each transcript: DN100 (100 nt immediately downstream of the transcript 3’ end), DN200 (200 nt, starting immediately downstream of DN100), and TRAN (spanning the entire transcript, except for the first and last 50 nt). Median expression values (normalized probe intensities) were calculated for each and a “readthrough score” equal to DN200 / TRAN was obtained for each gene in WT and *npl3Δ* strains. The readthrough scores obtained for the two strains were then used to calculate readthrough ratios, comparing readthrough in the *npl3*Δ mutant strain to that in WT yeast (S5 Table). A ratio greater than 1 indicates higher readthrough in the *npl3*Δ mutant strain. All mRNAs, snoRNAs, CUTs and SUTs were considered, with the exclusion of transcripts less than 200 nt in length, or closer than 400 nt to an annotated Ensembl feature on the same strand. S6A Fig shows the distribution of readthrough across all genes in the *npl3*Δ strain. The dark and light blue lines show the distribution of readthrough ratios for two replicate experiments, alongside the null ratio where WT is compared to WT (red). Strikingly, most genes show some level of readthrough in the *npl3Δ* strain. The number of genes showing significant readthrough (false discovery rate = 0.05) ranged from 29% (1165/3961) to 37% (1468/3961), depending on the experiment.

### Features of Npl3-regulated mRNAs

**Fig 7:**
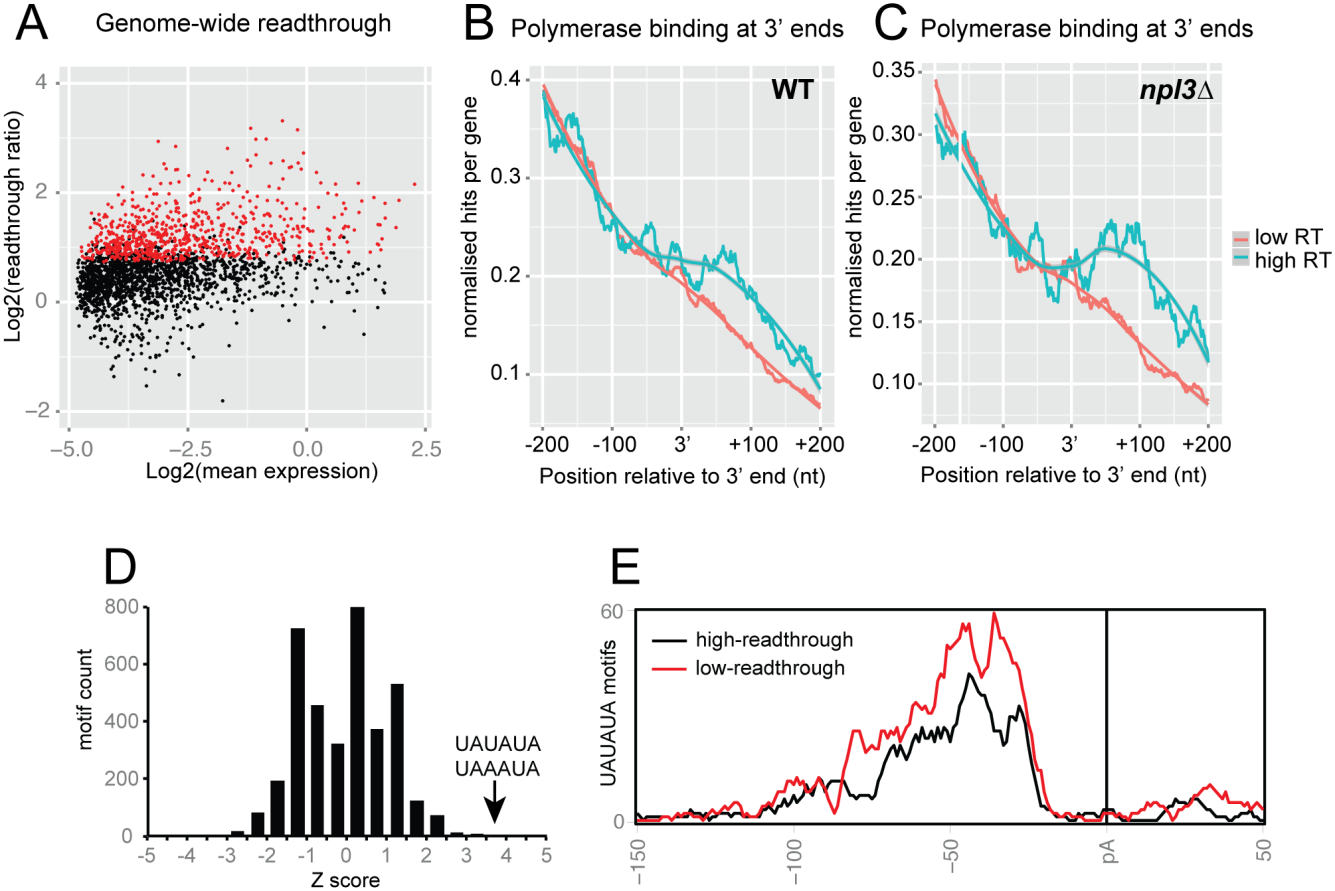
Features of readthrough genes. **A:** Scatterplot comparing readthrough and expression, including only those genes that passed the filters (see bioinformatics methods in supplementary information for description of filters). Points shown in red (709/2234) represent those genes showing significant readthrough in both experiments (FDR = 0.05). **B:** Polymerase occupancy around the 3' ends of 200 genes with the highest readthrough rank, and 1200 genes with the lowest rank. **C:** Polymerase occupancy around the 3' ends of genes, as in 7(B), measured in the *npl3*Δ background. **D:** Enrichment scores of 6-mer motifs in the 3’ region of low readthrough genes, relative to high readthrough genes. The 3’ region was defined as -80 to -20 nt from the polyadenylation site [37]. Low and high readthrough genes were defined as the bottom and top quartiles of mRNAs in the dataset. UAUAUA and UAAAUA are the most enriched 6-mer motif in low-readthrough genes. **E:** Localization of UAUAUA motifs around the polyadenylation sites of low readthrough genes (red) and high readthrough genes (black), aligned by the polyA site.

We applied stringent filters (see Bioinformatics section in Experimental Procedures) and plotted readthrough ratios for genes passing all filters (2234) against gene expression (Fig 7A); 32% of genes showed significant readthrough (marked red; FDR = 0.05), demonstrating a requirement for Npl3 in the termination of a substantial proportion of all RNAPII genes. We observed no clear correlation between readthrough ratio and expression level. We ranked all 2234 genes by readthrough ratio (S5 Table) and compared polymerase occupancy around the 3' ends of genes with the highest readthrough rank (top 200) and the control group with a low readthrough rank (1200 genes). We found that polymerase occupancy downstream of the 3' end is higher in high readthrough genes than low readthrough genes in WT yeast (Fig 7B). This suggests that these genes show a tendency towards readthrough, even in the presence of Npl3. This effect is more pronounced in the absence of Npl3 (Fig 7C), with a greater accumulation of polymerase downstream of the 3' end of high readthrough genes.

We next sought to identify factors that might discriminate high readthrough genes from low readthrough genes. We found that readthrough correlated weakly with gene length. Longer genes were more likely to show readthrough (S6B Fig), consistent with a report showing preferential binding of Npl3 to longer genes [6]. To identify potential motifs, we compared the 3’ regions from all genes in the top and bottom groups based on the readthrough ranking. This identified UAUAUA and UAAAUA motif as strongly over-represented in low readthrough genes (Fig 7D). UAUAUA is the binding site for the pre-mRNA 3’-end processing factor Hrp1 [62] and comparison of the locations of the UAUAUA motifs showed enrichment at the expected location upstream of the pA site in low readthrough genes (Fig 7E). The enrichment of Hrp1 binding sites in genes that do not show readthrough in the absence of Npl3 strongly suggests that direct, efficient recruitment of Hrp1 can bypass the requirement for Npl3 in termination.

Gene ontology analysis showed that genes with higher readthrough were enriched for plasma membrane proteins and functions in localization and/or transmembrane transport (S6 Table). This suggests these genes are potentially co-regulated through transcription termination.

## Discussion

### Apparent functions for Npl3 in surveillance and transcription termination

Npl3 is bound to all classes of RNAPII transcripts, with enrichment for oligoadenylated RNAs characteristic of nuclear surveillance targets. Deletion of *NPL3* revealed its involvement in termination on diverse transcripts that had not appeared to share termination systems. These included many mRNAs and ncRNAs including the CUT class of lncRNAs and most snoRNAs. In contrast, no defects were seen for snRNAs, which have 3’ processing and termination pathways that appeared to closely resemble snoRNAs.

Significant transcription termination defects were seen on approximately 30% of protein coding genes in *npl3Δ* strains. Readthrough was associated with widespread gene expression changes due to transcriptional interference at downstream genes. This likely reflects the disruption of nucleosome positioning and/or transcription factor binding caused by passage of RNAPII through the nucleosome free regions characteristic of yeast promoters. The precise number of genes that are inhibited by this mechanism is difficult to determine accurately. In the cases of the convergent genes highlighted in the text, the phenotype is clear because the transcripts lie on opposite strands. However, actively transcribed, convergent genes are quite rare in yeast, and transcriptional interference on tandem genes may be less evident. Downstream gene expression may appear unaffected on microarrays, despite generating little functional mRNA, with downstream signal representing extended upstream gene products. From the RNAPII CRAC data it appears that sense-orientated genes some distance from a site of readthrough can display the hallmarks of decreased expression. This was shown, for example, by the decreased RNAPII peak at the 5’ end of the *HOR2* gene, located downstream of the extended *VHR2* transcript (Fig 3).

The widespread interference seen in the absence of Npl3 highlights the necessity for very efficient release of RNAPII at the 3’ ends of genes. In general, fold changes in RNAPII occupancy were less marked than changes in downstream transcript levels. This indicates that readthrough by a small number of polymerases can drastically alter the regulation of gene-expression. In the case of snoRNAs, it appears that low levels of transcription readthrough, as determined by accumulation of downstream RNAPII, result in high levels of extended transcripts. Normal snoRNA termination and processing require the NSS complex, which stimulates exosome recruitment [27], and Nab3 association with snoRNAs was reduced in *npl3Δ* strains. These observations strongly indicate that loss of Npl3 also leads to defects in snoRNA 3’ processing and/or surveillance of 3’ extended species. The relative contributions of impaired snoRNA processing versus impaired surveillance in *npl3Δ* mutants is difficult to assess—as is the case for many substrates for nuclear surveillance/processing factors. Distinguishing the contributions of processing and surveillance is not generally feasible when the phenotype is accumulation of extended species at steady state, and will require the development of very fast, *in vivo* kinetic analyses.

### Potential roles for Npl3 in termination

Termination defects seen in the absence of Npl3 were restricted to RNAPII. However, while diverse classes of RNAPII transcripts are affected, this was not the case for all transcripts of any class. To try to understand what determines this apparent variability in the requirement for Npl3, we ranked protein-coding genes by their degree of readthrough (readthrough ratio) in the absence of Npl3, and sought correlated features in protein coding genes.

A notable correlation was with the elevated presence of consensus, UAUAUA binding sites for the mRNA 3’ cleavage factor Hrp1 in the 3’ regions of transcripts with low readthrough scores (i.e. with low dependence on Npl3 for termination). We postulate that association of Hrp1 and/or other cleavage factors with the pre-mRNA is normally promoted by Npl3-mediated packaging, but this requirement can be alleviated by the presence of high-affinity RNA-binding sites. In contrast, competition between binding of Npl3 and pre-mRNA cleavage and polyadenylation factors including Hrp1 was previously reported for *GAL* reporter constructs [7,11]. This apparent anti-termination activity of Npl3 is the opposite of our general findings. However, it could readily be envisaged that on individual genes, Npl3 binding sites conflict with the association of specific factors. The *GAL* genes are not expressed under the conditions used in our analyses, making it difficult to determine whether these effects are also seen on the endogenous genes.

Readthrough ratio was weakly correlated with gene length, with longer genes more likely to exhibit termination defects when Npl3 was absent. Preferential association of Npl3 with longer transcripts as been reported [6], suggesting that these may show greater changes in pre-mRNA packaging in its absence. However, we saw no clear length dependence for Npl3 in termination on ncRNAs, which are generally shorter than mRNAs.

Several distinct, but overlapping pathways for RNAPII termination are normally used by transcripts that are extended in the absence of Npl3. On pre-mRNAs, recognition of the cleavage and polyadenylation site is linked to changes in the transcribing polymerase that make it prone to termination at downstream pause sites. This may involve Tyr1 dephosphorylation in the CTD by the Glc7 phosphatase that associates with the CPF-CF [63]. Loss of Tyr1P promotes binding of the cleavage factor Pcf11, as well as Rtt103, which in turn recruits the Rai1/Rat1 complex for the “torpedo” termination pathway. In contrast, termination of a wide range of ncRNA transcripts involves the Nrd1/Nab3/Sen1 (NNS) complex, which binds to the nascent transcript and to the RNAPII CTD with Ser5P modification, as well as the TRAMP nuclear surveillance complex and promoter proximal nucleosomes with H3K4 trimethylation ([18,22,34,36,59] reviewed in [64]). Other termination mechanisms are initiated by co-transcriptional cleavage by the RNase III homologue Rnt1 [52,65] and by formation of a transcription elongation “roadblock” due to Reb1 binding on the DNA [66].

We found no correlation between known Rnt1 or Reb1 targets and transcription readthrough in *npl3*Δ strains. Binding of Nab3 to the CUT lncRNAs was increased in *npl3*Δ strains. A simple, potential explanation might be that the absence of the, normally very abundant, Npl3 protein frees binding sites that can be occupied by other factors, including Nrd1-Nab3. However, the abundance and readthrough of CUTs were also increased in the absence of Npl3, and this may contribute to the apparent changes in Nab3 association. We propose that loss of Npl3 results in aberrant RNP formation that still permits Nab3 recruitment, but binding may be non-productive.

Npl3 was reported to directly stimulate RNAPII elongation and a mutant that disrupts this function, *npl3-120*, resulted in improved termination. The slower RNAPII elongation rate in *npl3-120* strains may enhance termination by increasing the time available for recruitment of 3' end processing factors such as Hrp1. In contrast, an Npl3 mutant (S411A) that blocks a phosphorylation site was associated with impaired transcription termination [67]. This defect was proposed to arise from retention of the mutant Npl3 in association with the RNAPII CTD and the mRNA. However, the list of genes showing 3’ extension in Npl3S_411_A strains overlaps substantially with the genes showing RT in *npl3*Δ, indicating that Npl3 retention is not solely responsible for this phenotype. Of the 818 genes showing 3’ extension in Npl3S_411_A, 143 overlap with the 614 genes showing significant readthrough in *npl3*Δ (p-value 7.1e-16, Fisher’s exact test).

Npl3 is a highly abundant RNA binding protein that participates in many processing events and associates with all nascent RNAPII transcripts. It seems probable that its absence will result in substantial changes in the nascent RNP structure. We speculate that such inappropriately packaged RNA is associated with downstream defects in transcription termination, reflected by changes in binding by the termination factor Nab3, consequently impairing a remodeling event that promotes removal of the polymerase from the nascent transcript.

## Materials and Methods

### Strains and media

Yeast were grown in standard SD medium at 30°C unless otherwise stated. Strains and plasmids used are listed in S7 Table.

### Oligonucleotides

All oligonucleotides used are listed in S8 Table.

### Strain construction

All yeast analyses were performed in strains derived from BY4741 (*MAT*a; *his3Δ1*; *leu2Δ0*; *met15Δ0*; *ura3Δ0*) or, in the case of N-PTH-NPL3, BY4727 (*MATalpha*; *his3Δ200*; *leu2Δ0*; *lys2Δ0*; *met15Δ0*; *trp1Δ63*; *ura3Δ0*). N-PTH-NPL3 is a strain in which a sequence encoding a PTH (proteinA-TEV-His) tag was integrated at the 5' end of *NPL3*, resulting in the formation of an N-terminally tagged protein utilizing the endogenous *NPL3* promoter. As the protein is N-terminally tagged in this strain, the orientation of the tag is reversed, allowing the order of protein purification steps to be retained. Generation of this strain involved inserting a *URA3* marker between the *NPL3* promoter and the *NPL3* ORF, and then replacing the *URA3* marker with a sequence encoding the PTH tag. The second PCR, amplifying the PTH tag, was performed on a plasmid expressing N-PTH-NPL3 (pRS415-NPL3-PTH), and amplified a region running from the start of the PTH tag to ~600 nt into the *NPL3* ORF to increase integration efficiency.

### Crosslinking and analyses of cDNAs (CRAC)

The CRAC procedure involves purifying protein/RNA complexes, where the RNA has been covalently UV crosslinked to the protein [41]. RNA-protein complexes are purified, and RNAs are partially digested to leave only the 'footprint' bound by the protein. Linkers are then ligated to both ends and the protein is removed by proteinase K digestion. RNAs are reverse transcribed and resulting cDNAs subjected to next generation sequencing using the Illumina platform (Edinburgh Genomics).

### Strand-specific tiling arrays

WT and *npl3Δ* yeast were grown to mid-log phase (OD_600_ ~0.5) and cells were collected by brief centrifugation (3000 x*g*, for 5 min). Total RNA was isolated by a standard acidic hot phenol method and DNA was removed by treating with RNase-free DNaseI (Turbo DNA-free kit; Ambion). Reverse transcription and array hybridizations were carried out as previously described [68].

### RT-qPCR

Yeast cultures were grown to mid-log phase (OD_600_ ~0.5) and cells were collected by brief centrifugation (3000 xg, for 5 minutes). Total RNA was isolated by a standard acidic hot phenol method and DNA was removed by treating with RNase-free DNaseI (Turbo DNA-free kit; Ambion). Single stranded cDNA was generated using gene specific primers, designed to prime from the 3' end of the transcript (to measure expression) or from ~500 nt downstream (to measure transcriptional readthrough). Reverse transcription reactions were performed using Superscript III (Invitrogen). The expression level of individual transcripts was determined by quantitative PCR using SYBR green fluorescence for detection. Relative quantities were calculated using a standard curve made with known concentrations of genomic DNA, and were normalized to levels of *ACT1* in each RNA sample.

### Northern blotting

Total RNA was isolated by a standard acidic hot phenol method. For SNR60 readthrough analysis, equal amounts of RNA (10 μg) were resolved on a 1.2% agarose gel in TBE buffer and transferred onto Hybond N+ nitrocellulose membrane overnight in 6x SSC. For detection of mature snoRNAs and RNase H cleavage assay products, samples (4 μg total RNA for snoRNA detection) were resolved on an 8% acrylamide gel containing 8.3 M urea, in TBE buffer and transferred onto Hybond N+ nitrocellulose overnight in 0.5x TBE. Oligo probes were end labeled with [γ-^32^P] ATP and hybridized to the membrane overnight at 37°C in ULTRAhyb-Oligo (Ambion). Signals were detected using a Fuji FLA-5100.

### RNase H treatment

Samples (30 μg) of RNA were annealed with 750 ng oligo-dT and/or 10 pMoles gene-specific oligo, heated to 65°C and allowed to cool slowly to 30°C. Samples were then incubated with 1 unit RNase H (Roche) at 30°C for 1 hour.

### Western blotting

Total extract from crosslinked CRAC samples were loaded onto 4–12% NuPAGE gels and Transferred onto Hybond C nitrocellulose membrane. Following blocking in 5% milk, the membrane was incubated first inn anti-TAP primary antibody (1:5000 overnight) and then anti-rabbit secondary (1:10000 for 1 hour). Signal was visualized using the Licor Odyssey system.

### Sequencing data

All sequence data are available from GEO under accession number GSE70191.

http://www.ncbi.nlm.nih.gov/geo/query/acc.cgi?token=gdivgqmivxcpzkp&acc=GSE70191

Sequencing data were processed and quality filtered using the fastx toolkit as previously described [37]. Processed reads were mapped to the *Saccharomyces cerevisiae* genome (SGD v64) using Novoalign (Novocraft) with genome annotation from Ensembl (EF4.74), supplemented with non-coding sequences as previously described [37]. Reads mapping to different transcript RNA classes were determined using the pyCRAC package [17] (Figs 1A, 1C, S3B and S5B). All analyses were performed using genome SGD v64 unless otherwise stated. The distribution of hits across transcripts of different classes was determined in several ways. Firstly, to examine the distribution of proteins at the 5' and 3' ends of mRNAs, hits within 300–900 nt windows aligned to the start (TSS) and end (pA) were plotted using published scripts [37]. The top 2000 bound mRNAs for each protein were included in the analysis and average distribution was plotted (Fig 1B and 1D). A similar analysis was performed to assess binding at snoRNAs and CUTs (Figs 1E, 1F, 4D, 5A, 6B and 6C). In this instance smaller windows were used and hits per million mapped reads were plotted, rather than average distribution. Reads were aligned to the TSS or 3' ends, with flanking regions included as shown. We included all snoRNAs in the analysis, but only included CUTs > 150 nt in length. Hits at introns were also plotted using this approach (S1C and S1D Fig). As an alternative way to assess binding across transcripts, we used pyBinCollector from the pyCRAC package, which normalizes transcripts by length, dividing hits into a given number of bins (S1E, S1F, S3C, S3D, S5C and S5D Figs). Rpo21 occupancy was calculated to determine transcriptional readthrough (Figs 3C, 4C, 5D, S2C and S4C) using pyPileup from the pyCRAC package, with default settings. Hits containing unencoded 3' oligoA tails of 2 of more were determined using a reported pipeline [37,69]. These hits were then mapped to transcript groups and plotted across RNA classes as described above (Fig 1C and 1D).

### Tiling arrays

All microarray data are available in the ArrayExpress database (http://www.ebi.ac.uk/arrayexpress), under accession number E-MTAB-3642. Array data can also be visualized in a genome browser heat map format (http://steinmetzlab.embl.de/tollerveyLabArray). Microarray data were aligned to SGD *S*.*cerevisiae* genome version (SGD v57). Normalization of microarray hybridizations was performed as previously described [70] and transcript boundaries shown are as published [71]. Differential expression analyses were carried out using the R-package, Limma [72], controlling for the false discovery rate arising from multiple testing [73]. Five snoRNAs were not included in the differential expression analyses due to lack of transcript boundary information (Fig 2 and S5 Table). These can, however, be viewed in the genome browser heat map.

### Aligning PolII CRAC data with array data

CRAC hit data were aligned to SGD *S*.*cerevisiae* genome version (SGD v57) alongside tiling array expression data at individual loci (Figs 3A, 4A, 4B, 5B, S2A and S4A). Hits were normalized for library size by plotting hits per million mapped reads at each nucleotide. Fig 6A shows CRAC data aligned to SGD v57 without array data.

### Analysis of genome-wide transcriptional readthrough

Readthrough scores were calculated for mono-exonic snoRNAs, mRNAs, CUTs and SUTs with coordinates previously defined [71]. Transcripts that are < 200 nt were excluded, as were transcripts < 400 bp upstream of another annotated transcript [71] or in Ensembl release 68. The exception to this is when an mRNA has an annotated CUT or SUT immediately downstream of the 3' end. In some instances, these annotated ncRNAs appear to correspond to upstream mRNA readthrough, and therefore these mRNAs were not filtered out. Three windows were defined for each transcript: DN100 (100 nt immediately downstream of the transcript 3’ end), DN200 (200 nt, starting immediately downstream of DN100), and TRAN (spanning the entire transcript, except for the first and last 50 nt). The median normalized probe intensities (in log2 space) for each microarray sample were calculated for each window, although windows with < 8 probes were excluded.

The readthrough score was then defined for each gene and each sample (wild-type replicate 1, wild-type replicate 2, npl3-delta replicate 1, and npl3-delta replicate 2) as the median intensity for DN200, minus the median intensity for TRAN. The difference in npl3-delta and wild-type transcriptional readthrough was determined by calculating a readthrough ratio for each gene, defined as the readthrough score for npl3-delta minus the readthrough score for wild-type. Readthrough ratios were also calculated for wild-type replicate 2 versus wild-type replicate 1, to provide an empirical null distribution and enable transcripts with a significant increase in readthrough for npl3-delta versus wild-type to be identified. The Benjamini–Hochberg procedure was used to control the false discovery rate at 0.05. For this step, the two replicate experiments were treated separately, then a stringent list of genes with elevated readthrough obtained by intersecting the results from both replicates.

A series of filters was used to exclude transcripts for which readthrough ratios may be inaccurate, either due to low expression or because of evidence of independent transcription initiation downstream. The following criteria were used: (i) there must be < 10 Cbc1 (cap-binding complex protein 1) CRAC reads in the DN100 window, (ii) TRAN median probe intensity must be > -4.88 for wild-type and npl3-delta, (iii) for npl3-delta, the median probe intensity in the DN100 window must be > 70% that of the TRAN window, and (iv) the median probe intensity in the TRAN window for npl3-delta must be at least 70% that of the same window in the wild-type sample. For filters (ii)-(iv), the mean of the two replicates was used.

Plots of Pol II distribution in regions centered on transcript 3’ ends were obtained by taking the individual Pol II CRAC read distributions for each gene, linearly transforming each gene so that its maximum value was equal to 1, and then summing at each nucleotide for the indicated set of genes (either high or low readthrough groups). We observed that genes with the very lowest readthrough ranks had a spurious negative readthrough ratio due to having increased expression in the *npl3*Δ strain relative to WT. To limit the contribution of these genes, we took a larger number of genes for the low readthrough group (1200 compared to 200).

### Motif analyses

Npl3 binding sites were analyzed for enriched motifs by first filtering total reads to exclude low complexity sequences, as previously described [37]. The pyCRAC package [17] was used to calculate statistical overrepresentation scores for every possible k-mer (S1D Fig) using a previously described algorithm [69]. We used pyCRAC to calculate False Discovery rates (FDRs) and selected only reads forming clusters of 5 reads or more with an FDR < 0.05 for further analysis. Reads were further filtered to include only those with one or more T-C substitution, representing a site of crosslinking, and therefore predicted to indicate genuine binding sites with greater stringency.

To identify sequence motifs that differentiate high- and low-readthrough genes, we considered the 1822 genes for which reliable readthrough scores could be established, and separated these genes into quartiles by their readthrough scores. 2234 genes were included in the genome-wide readthrough analysis, but the bottom 250 were excluded from the motif analyses as these were found to have spuriously low readthrough ratios resulting from increased expression in the *npl3*Δ mutant. Of the remaining 1984 genes, only those with well-defined polyA sites (1822) were included in the motif analysis. For each 6-mer nucleotide motif, we calculated the numbers of genes in each quartile that contained the motif within the region (-80 to -20 nucleotides) from the polyadenylation site (polyA site). The polyA site was defined from Pab1 CRAC data as described [37]. We then identified the motifs that were significantly enriched in the low-readthrough genes, relative to high-readthrough genes, by calculating Z-scores as described [69]. To illustrate the localization of motifs relative the polyA site, we plotted the total coverage of UAUAUA motifs as a function of distance from the polyA site, separately for the top and bottom quartile of genes ranked by readthrough scores.

## Supporting Information

S1 Fig Npl3 RNA binding profile. **A:** Drop test comparing the growth of WT, PTH-Npl3 and npl3Δ at 30°C and 16°C. **B:** Autoradiogram showing Npl3 crosslinked to p32-labelled RNA (left panel). Right panel shows expression of PTH-tagged Npl3. **C:** Distribution of Npl3 across ribosomal protein gene (RPG) introns, aligned by intron 5' end (red line). **D:** Distribution of Npl3 across non-RPG introns, aligned by intron 5' end (red line). **E:** Top enriched motifs in Npl3-bounds mRNAs (k-mers = 4).
(PDF)

S2 Fig Transcriptional readthrough at the *UPF2-PTC7* locus. **A:** Expression (II-III) and polymerase occupancy (I, IV) at the *UPF2-PTC7* locus on chromosome 8, in WT and *npl3*Δ mutant yeast. Expression is determined using strand-specific tiling arrays, and two biological replicates are shown for both yeast strains (tracks labeled 1 and 2). Expression from the Watson strand is shown above the genomic co-ordinate information, and expression from the Crick strand is shown below. Polymerase occupancy on each strand is shown in blue (WT) or red (*npl3*Δ). **B:** Confirmation of transcriptional readthrough of *UPF2* (VHR2 RT) and down-regulation of *PTC7* in *npl3*Δ using strand- specific reverse transcription followed by qPCR. Approximate locations of primers used for reverse- transcription are shown in 4(A) (green arrows). *UPF2* readthrough is measured by performing a reverse transcription reaction using an oligo that primes from ~500 nt downstream from the *UPF2* 3' end. qPCR primers are located towards the 3’ end of *PTC7* and *UPF2*, respectively. The histogram shows change in the mutant, compared to WT. **C:** Comparison of tiling array expression data and polymerase occupancy at regions across the *UPF2-PTC7* locus, in WT and *npl3*Δ. Numbers represent change in the *npl3*Δ mutant relative to WT. **D:** Production of ‘normal’ polyadenylated UPF2 mRNA is reduced in *npl3*Δ. Samples were incubated with RNase H, UPF2-specific oligo (GSO; gene-specific oligo) with (lanes 2 and 5) or without (lanes 1 and 4) oligo-dT. Polyadenylated 3’ ends can be seen in lanes 1 and 4 in the WT and *npl3*Δ strains respectively. Lanes 1 and 4 show 3’ ends with the polyA tail removed.
(PDF)

S3 Fig Transcriptome-wide binding of polymerase II in WT and *npl3*Δ yeast. **A:** RNA binding (left) and expression (right) of HTP tagged Rpo21 in WT and *npl3*Δ yeast. **B:** Rpo21 binding across RNA classes in WT and *npl3Δ* yeast.
(PDF)

S4 Fig Transcriptional read-through of *SNR3* in *npl3*Δ yeast. **A:** Expression and polymerase occupancy at the *SNR3/EFM3* locus, in WT and *npl3*Δ mutant yeast. Expression is determined using strand-specific tiling arrays, and two biological replicates are shown for both yeast strains (tracks labeled 1 and 2). Expression from the Watson strand is shown above the genomic co-ordinate information, and expression from the Crick strand is shown below. Polymerase occupancy on each strand is shown in blue (WT) or red (*npl3*Δ). **B:** Confirmation of transcriptional readthrough of *SNR3* and down-regulation of *EFM3* in *npl3*Δ using strand- specific reverse transcription followed by qPCR. The histogram shows changes in the *npl3Δ* mutant, compared to the WT levels (which were set to 1). **C:** Comparison of tiling array expression data and polymerase occupancy at regions across the *SNR3* locus, in WT and *npl3*Δ. Numbers represent change in the *npl3*Δ mutant relative to WT. **D:** Northern blot to detect abundance on mature snoRNAs in WT and *npl3*Δ (2 biological replicates).
(PDF)

S5 Fig Transcriptome-wide binding of Nab3 in WT and *npl3*Δ. **A:** RNA binding (left) and expression (right) of HTP tagged Nab3 in WT and *npl3*Δ yeast. **B:** Nab3 binding across RNA classes in WT and *npl3Δ* yeast, 2 biological replicates per strain.
**C**: Metagene analysis of Nab3 binding across mRNAs. Average distribution of Nab3 around the 5’ and 3’ ends of mRNAs in WT (blue) and *npl3*Δ (red). Transcripts are aligned at the transcription start sites (TSS) and polyA (pA) site. Pale dots depict precise number of hits at particular nucleotide positions and the darker colours show lines of best fit. Hits are normalized to a total of 1 across all mRNAs.
(PDF)

S6 Fig Genome-wide transcriptional readthrough. **A:** Distribution of readthrough ratios. The red line shows the null ratio, which is the comparison of two biological replicates of readthrough in WT yeast. The light and dark blue lines show readthrough ratios of *npl3*Δ: WT, comparing two biological replicates. For the first experiment, 1468 genes showed significant readthrough, and for the second experiment this number is 1165 (FDR = 0.05). This plot shows the distribution of readthrough for all mRNAs, snoRNAs, CUTs and SUTs, excluding those that are less than 200 nt in length or less than 400 nt from an annotated Ensembl feature on the same strand (total 3961). **B:** Scatterplot showing readthrough ratio against gene length. The linear regression line is shown in red. Spearman's correlation ρ = 0.21, p-value <2.2e-16.
(PDF)

S1 Table Transcript expression levels. Table includes expression data for coding and non-coding transcripts, derived from normalized probe intensity data. Log2 expression data are shown for two biological replicates of WT (wt_1, wt_2) and *npl3*Δ mutant (npl3Δ_1, npl3Δ_2).
(XLSX)

S2 Table Differential expression analysis. Differences in gene expression between WT and *npl3Δ* yeast were determined using Limma. Those with significantly altered expression (adj p-value <0.05) show TRUE in column L (threshold).
(XLSX)

S3 Table mRNAs over-represented in *npl3*Δ. The 30 mRNAs most over-represented in *npl3*Δ relative to WT are listed by expression change (log2 fold change in mutant relative to WT).
(PDF)

S4 Table snoRNA readthrough. Table lists every snoRNA with information on class, transcript type, requirement for Rnt1 processing and presence of transcriptional readthrough.
(XLSX)

S5 Table Genome-wide readthrough. Table lists all transcripts included in readthrough analysis, with the corresponding readthrough scores (RT) and readthrough ratios.
(XLSX)

S6 Table GO term analysis. The GOrilla gene ontology and enrichment analysis visualization tool (http://cbl-gorilla.cs.technion.ac.il) was used to search for enriched gene ontology terms in a list of mRNAs ranked by readthrough ratio [74,75]. Top GO terms in each category are listed alongside respective significance.
(PDF)

S7 Table Strains and plasmids. (PDF)

S8 Table Oligonucleotides. (PDF)
