# Supplementary figures and images for: Loss of the Yeast SR Protein Npl3 Alters Gene Expression Due to Transcription Readthrough
Source: PLoS Genet. 2015 Dec 22;11(12):e1005735. doi: 10.1371/journal.pgen.1005735 (PMC4687934; doi:10.1371/journal.pgen.1005735)

A

30°C

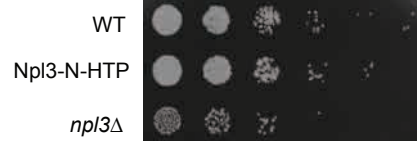

16°C

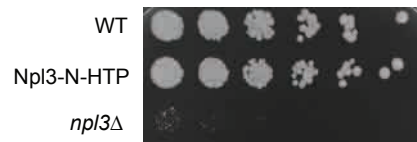

B

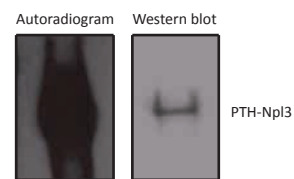

C

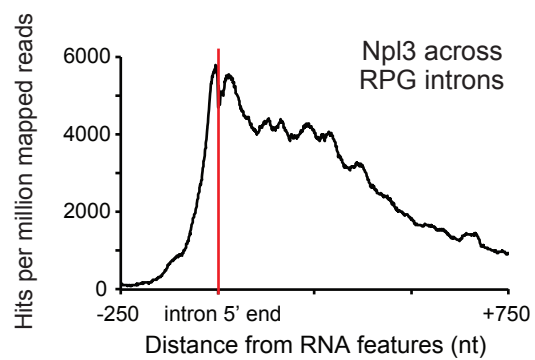

D

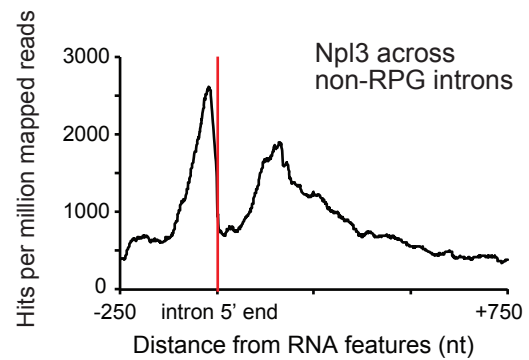

E

| k-mer | z-score |
|-------|---------|
| UGAG  | 12.72   |
| AUUG  | 10.95   |
| UCUG  | 10.12   |
| AGUG  | 10.01   |

Supplement: S1 Fig — A: Drop test comparing the growth of WT, PTH-Npl3 and npl3Δ at 30°C and 16°C. B: Autoradiogram showing Npl3 crosslinked to p32-labelled RNA (left panel). Right panel shows expression of PTH-tagged Npl3. C: Distribution of Npl3 across ribosomal protein gene (RPG) introns, aligned by intron 5' end (red line). D: Distribution of Npl3 across non-RPG introns, aligned by intron 5' end (red line). E: Top enriched motifs in Npl3-bounds mRNAs (k-mers = 4). (PDF) [file pgen.1005735.s001.pdf]

**A**

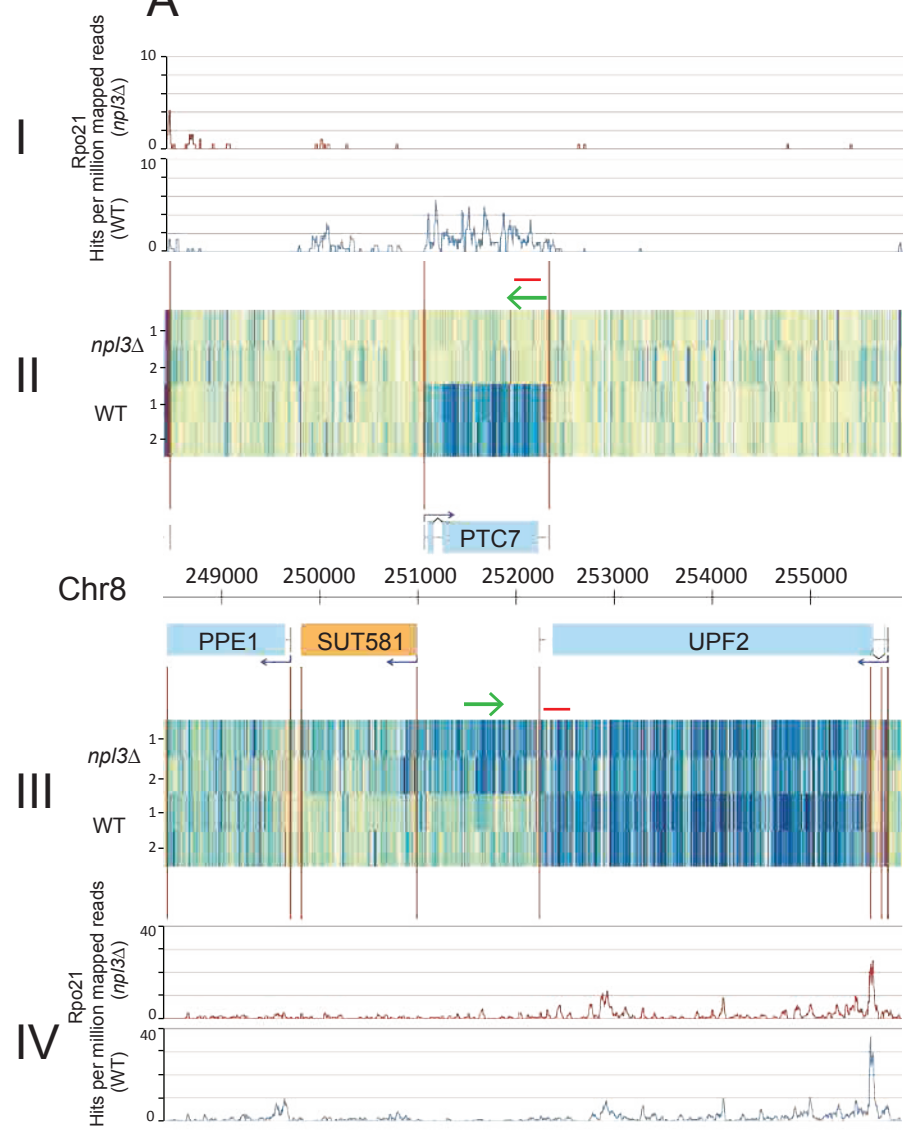

**B**

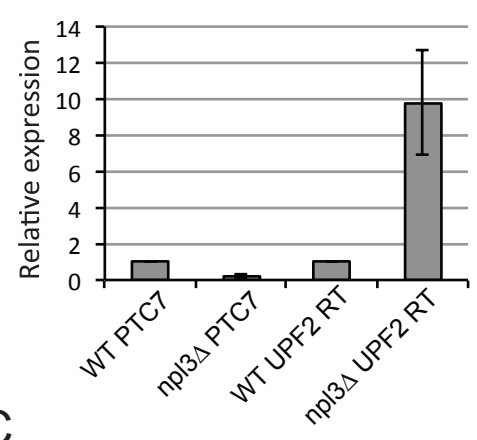

**C**

|             | Expression $np/3\Delta$ /WT | PollI $np/3\Delta$ /WT |
|-------------|-----------------------------|------------------------|
| UPF2        | 0.7                         | 0.8                    |
| UPF2-SUT581 |                             | 1.8                    |
| SUT581      | 2.1                         | 0.5                    |
| PPE1        | 0.9                         | 0.4                    |
| PTC7        | 0.1                         | 0.0                    |

**D**

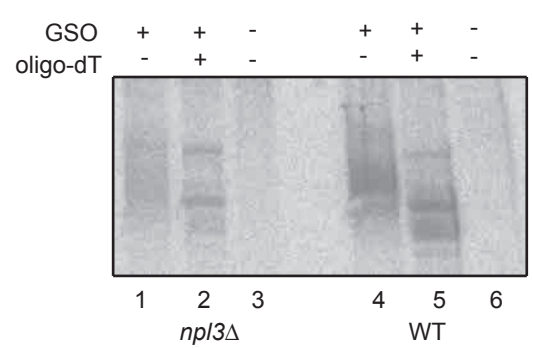

Supplement: S2 Fig — A: Expression (II-III) and polymerase occupancy (I, IV) at the UPF2-PTC7 locus on chromosome 8, in WT and npl3Δ mutant yeast. Expression is determined using strand-specific tiling arrays, and two biological replicates are shown for both yeast strains (tracks labeled 1 and 2). Expression from the Watson strand is shown above the genomic co-ordinate information, and expression from the Crick strand is shown below. Polymerase occupancy on each strand is shown in blue (WT) or red (npl3Δ). B: Confirmation of transcriptional readthrough of UPF2 (VHR2 RT) and down-regulation of PTC7 in npl3Δ using strand- specific reverse transcription followed by qPCR. Approximate locations of primers used for reverse- transcription are shown in 4(A) (green arrows). UPF2 readthrough is measured by performing a reverse transcription reaction using an oligo that primes from ~500 nt downstream from the UPF2 3' end. qPCR primers are located towards the 3’ end of PTC7 and UPF2, respectively. The histogram shows change in the mutant, compared to WT. C: Comparison of tiling array expression data and polymerase occupancy at regions across the UPF2-PTC7 locus, in WT and npl3Δ. Numbers represent change in the npl3Δ mutant relative to WT. D: Production of ‘normal’ polyadenylated UPF2 mRNA is reduced in npl3Δ. Samples were incubated with RNase H, UPF2-specific oligo (GSO; gene-specific oligo) with (lanes 2 and 5) or without (lanes 1 and 4) oligo-dT. Polyadenylated 3’ ends can be seen in lanes 1 and 4 in the WT and npl3Δ strains respectively. Lanes 1 and 4 show 3’ ends with the polyA tail removed. (PDF) [file pgen.1005735.s002.pdf]

A

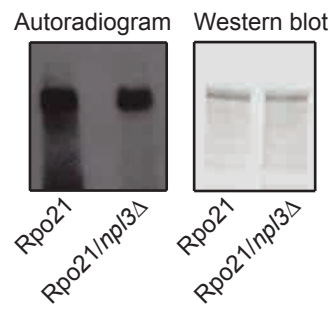

B

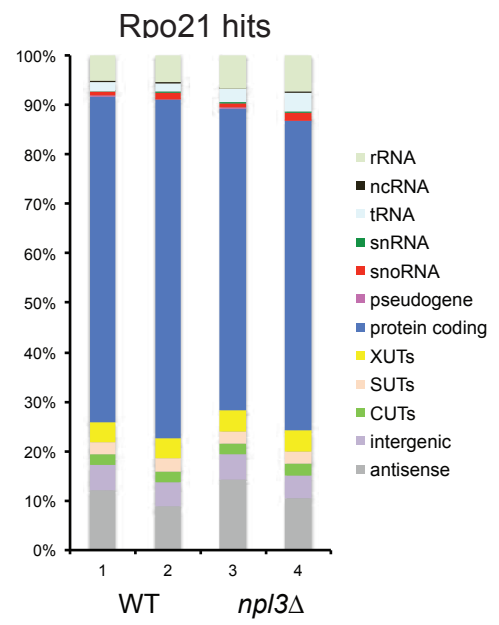

Supplement: S3 Fig — A: RNA binding (left) and expression (right) of HTP tagged Rpo21 in WT and npl3Δ yeast. B: Rpo21 binding across RNA classes in WT and npl3Δ yeast. (PDF) [file pgen.1005735.s003.pdf]

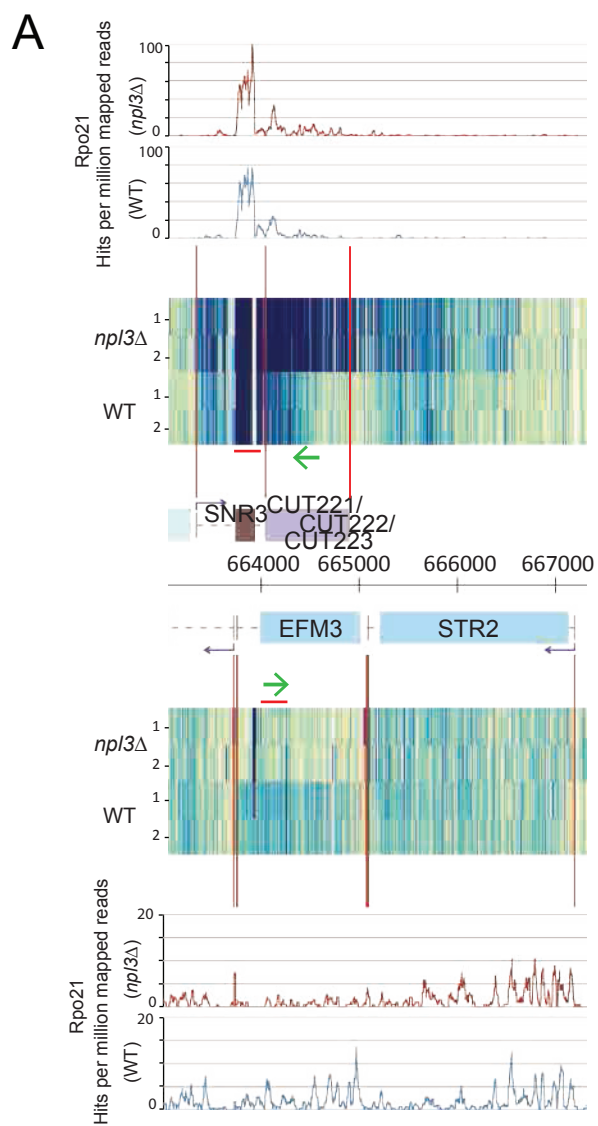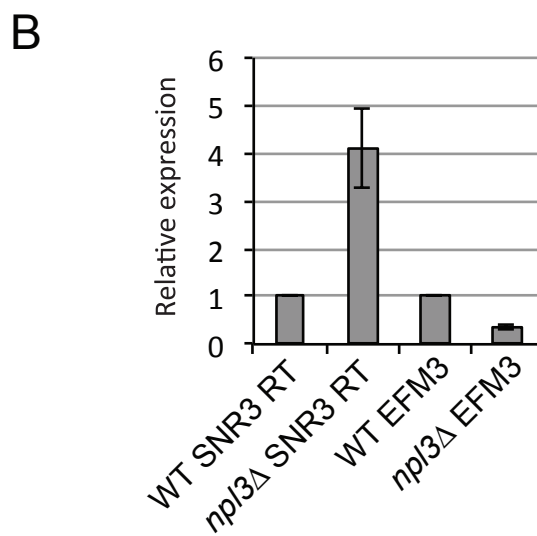

**C**

|        | Expression<br><i>np13Δ</i> /WT | PolIII<br><i>np13Δ</i> /WT |
|--------|--------------------------------|----------------------------|
| SNR3   | 4.6                            | 1.1                        |
| CUT221 | 5.6                            | 1.3                        |
| CUT222 | 21.6                           | 2.5                        |
| CUT223 | 29.4                           | 7.0                        |
| EFM3   | 0.2                            | 0.4                        |

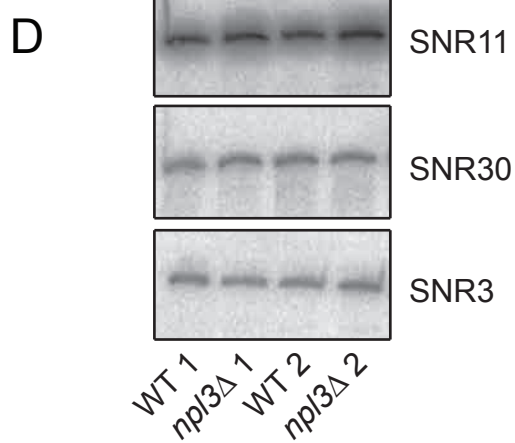

Supplement: S4 Fig — A: Expression and polymerase occupancy at the SNR3/EFM3 locus, in WT and npl3Δ mutant yeast. Expression is determined using strand-specific tiling arrays, and two biological replicates are shown for both yeast strains (tracks labeled 1 and 2). Expression from the Watson strand is shown above the genomic co-ordinate information, and expression from the Crick strand is shown below. Polymerase occupancy on each strand is shown in blue (WT) or red (npl3Δ). B: Confirmation of transcriptional readthrough of SNR3 and down-regulation of EFM3 in npl3Δ using strand- specific reverse transcription followed by qPCR. The histogram shows changes in the npl3Δ mutant, compared to the WT levels (which were set to 1). C: Comparison of tiling array expression data and polymerase occupancy at regions across the SNR3 locus, in WT and npl3Δ. Numbers represent change in the npl3Δ mutant relative to WT. D: Northern blot to detect abundance on mature snoRNAs in WT and npl3Δ (2 biological replicates). (PDF) [file pgen.1005735.s004.pdf]

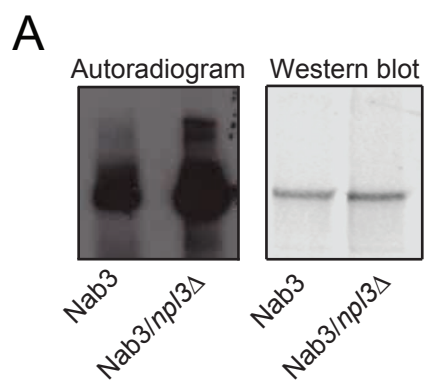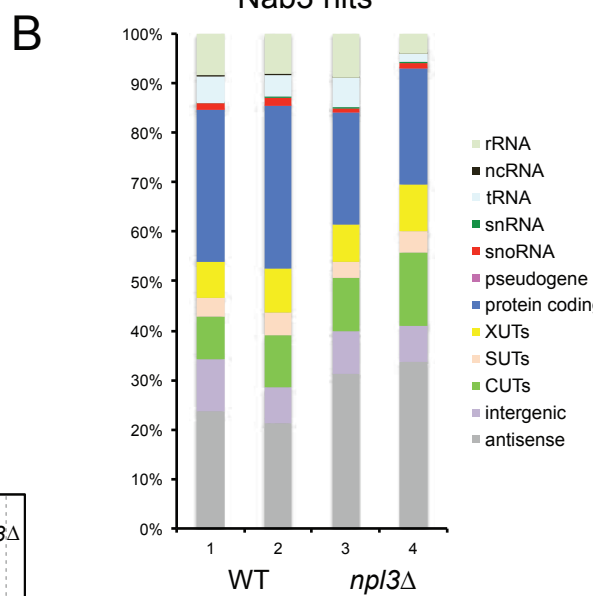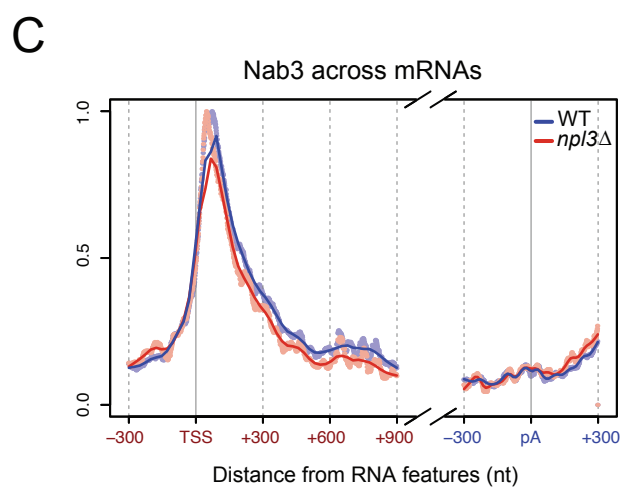

Supplement: S5 Fig — A: RNA binding (left) and expression (right) of HTP tagged Nab3 in WT and npl3Δ yeast. B: Nab3 binding across RNA classes in WT and npl3Δ yeast, 2 biological replicates per strain. C: Metagene analysis of Nab3 binding across mRNAs. Average distribution of Nab3 around the 5’ and 3’ ends of mRNAs in WT (blue) and npl3Δ (red). Transcripts are aligned at the transcription start sites (TSS) and polyA (pA) site. Pale dots depict precise number of hits at particular nucleotide positions and the darker colours show lines of best fit. Hits are normalized to a total of 1 across all mRNAs. (PDF) [file pgen.1005735.s005.pdf]

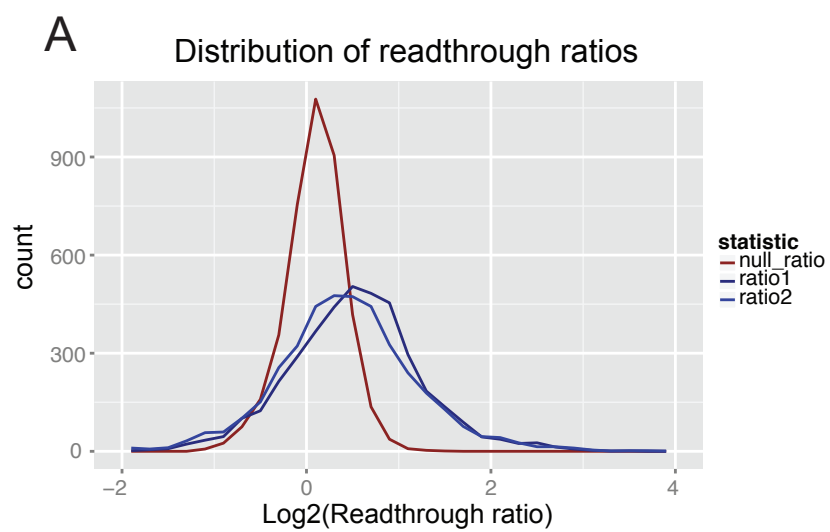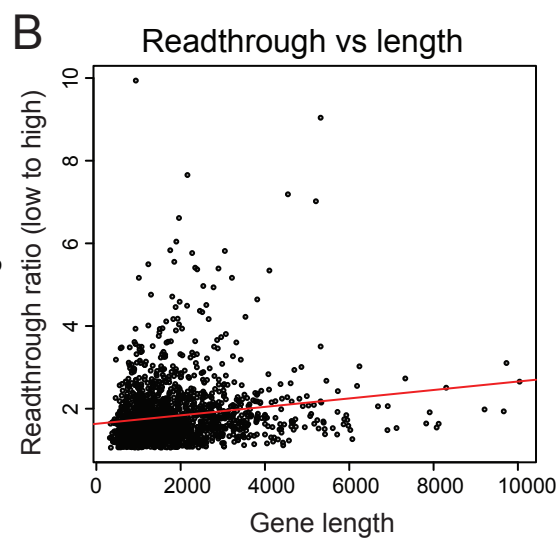

Supplement: S6 Fig — A: Distribution of readthrough ratios. The red line shows the null ratio, which is the comparison of two biological replicates of readthrough in WT yeast. The light and dark blue lines show readthrough ratios of npl3Δ: WT, comparing two biological replicates. For the first experiment, 1468 genes showed significant readthrough, and for the second experiment this number is 1165 (FDR = 0.05). This plot shows the distribution of readthrough for all mRNAs, snoRNAs, CUTs and SUTs, excluding those that are less than 200 nt in length or less than 400 nt from an annotated Ensembl feature on the same strand (total 3961). B: Scatterplot showing readthrough ratio against gene length. The linear regression line is shown in red. Spearman's correlation ρ = 0.21, p-value <2.2e-16. (PDF) [file pgen.1005735.s006.pdf]
