# Supplementary material for: Loss of the Yeast SR Protein Npl3 Alters Gene Expression Due to Transcription Readthrough
Source: PLoS Genet. 2015 Dec 22;11(12):e1005735. doi: 10.1371/journal.pgen.1005735 (PMC4687934; doi:10.1371/journal.pgen.1005735)
Supplement: S3 Table — The 30 mRNAs most over-represented in npl3Δ relative to WT are listed by expression change (log2 fold change in mutant relative to WT). (PDF) [file pgen.1005735.s009.pdf]

Supplementary Table S3

Gene	Expression level[a]	Local transcription changes in mutant	Category
RPL13A	3.32	Increased intron signal	intron
FLX1	3.21	Upstream tandem RT	RT
RPL31A	2.87	Increased intron signal	intron
GRE1	2.56	Upstream tandem RT	RT
RPS21B	2.52	Increased intron signal	Intron
RPS30A	2.50	Increased intron signal	Intron
HSP12	2.49	UP	UP
INO1	2.36	Upstream tandem RT	RT
RPS18B	2.27	Increased intron signal	Intron
RPL13B	2.24	Increased intron signal	Intron
INO1	2.24	Upstream tandem RT	RT
DDR2	2.22	UP	UP
USE1	2.22	Upstream tandem RT	RT
RPS27A	2.14	Increased intron signal	Intron
RPL24A	2.13	Increased intron signal	Intron
YSP3	2.01	Upstream tandem RT	RT
AQY2	1.97	Upstream tandem RT	RT
YDR124W	1.94	UP	UP
HSP26	1.91	Upstream tandem RT	RT
PTH4	1.89	UP	UP
HES1	1.84	UP	UP
YPR145C-A	1.79	Upstream tandem RT	RT
RPS29A	1.78	Increased intron signal	Intron
RPL26B	1.74	Increased intron signal	Intron
ZPS1	1.72	Upstream tandem RT	RT
SUL2	1.69	Upstream tandem RT	RT
YNR062C	1.67	UP	UP
SWC5	1.65	UP	UP
RPL35B	1.63	Increased intron signal	Intron
IDP2	1.61	Upstream tandem RT	RT

[a] Expression determined as Log2 fold change in mutant relative to WT
