# Supplementary material for: Loss of the Yeast SR Protein Npl3 Alters Gene Expression Due to Transcription Readthrough
Source: PLoS Genet. 2015 Dec 22;11(12):e1005735. doi: 10.1371/journal.pgen.1005735 (PMC4687934; doi:10.1371/journal.pgen.1005735)
Supplement: S6 Table — The GOrilla gene ontology and enrichment analysis visualization tool (http://cbl-gorilla.cs.technion.ac.il) was used to search for enriched gene ontology terms in a list of mRNAs ranked by readthrough ratio [74,75]. Top GO terms in each category are listed alongside respective significance. (PDF) [file pgen.1005735.s012.pdf]

Supplementary Table S6

		p-value	FDR Q-value
Process			
	Transmembrane transport	9.55E-05	3.47E-01
	Single-organism cellular process	2.71E-04	4.93E-01
	Adenine salvage	4.92E-04	5.97E-01
Function			
	Transporter activity	2.17E-05	3.23E-02
	Glucosidase activity	3.36E-05	2.50E-02
	Catalytic activity	5.06E-05	2.51E-02
Component			
	Plasma membrane	7.11E-06	5.58E-03
	Intrinsic component of membrane	3.06E-04	1.20E-01
	Integral component of membrane	7.33E-04	1.92E-01

GO term analysis looking for enrichment of categories in a list of mRNAs ranked by readthrough (high to low)
