# Supplementary material for: Loss of the Yeast SR Protein Npl3 Alters Gene Expression Due to Transcription Readthrough
Source: PLoS Genet. 2015 Dec 22;11(12):e1005735. doi: 10.1371/journal.pgen.1005735 (PMC4687934; doi:10.1371/journal.pgen.1005735)
Supplement: S7 Table — (PDF) [file pgen.1005735.s013.pdf]

Supplementary Table S7: Strains and plasmids

**Strain**	**Genotype**	**Reference**
BY4741	*MAT*a; *his3Δ1*; *leu2Δ0*; *met15Δ0*; *ura3Δ0*	Brachmann et al., 1998
BY4727	*MATα*; *his3Δ200*; *leu2Δ0*; *lys2Δ0*; *met15Δ0*; *trp1Δ63*; *ura3Δ0*	Brachmann et al., 1998
N-PTH-NPL3	*MATα*; *his3Δ200*; *leu2Δ0*; *lys2Δ0*; *met15Δ0*; *trp1Δ63*; *ura3Δ0; NPL3-N-HTP*	This study
npl3Δ	*MAT*a; *his3Δ1*; *leu2Δ0*; *met15Δ0*; *ura3Δ0*; *npl3::KAN*	This study
Nab3-C-HTP	*MAT*a; *his3Δ1*; *leu2Δ0*; *met15Δ0*; *ura3Δ0*; *NAB3-HTP-URA3*	Wlotzka et al.,
Rpo21-C-HTP	*MAT*a; *his3Δ1*; *leu2Δ0*; *met15Δ0*; *ura3Δ0*; *RPO21-HTP-URA3*	Milligan et al., submitted
Nab3-C-HTP/npl3Δ	*MAT*a; *his3Δ1*; *leu2Δ0*; *met15Δ0*; *ura3Δ0*; *NAB3-HTP-URA3, npl3::NAT*	This study
Rpo21-C-HTP/npl3Δ	*MAT*a; *his3Δ1*; *leu2Δ0*; *met15Δ0*; *ura3Δ0*; *RPO21-HTP-URA3*, *npl3::NAT*	This study

**Plasmid**	**Genotype**	**Reference**
pRS415-NPL3-PTH	pRS415; N-terminally PTH tagged Npl3 under Met promoter	Granneman et al., 2009
