# Supplementary material for: Loss of the Yeast SR Protein Npl3 Alters Gene Expression Due to Transcription Readthrough
Source: PLoS Genet. 2015 Dec 22;11(12):e1005735. doi: 10.1371/journal.pgen.1005735 (PMC4687934; doi:10.1371/journal.pgen.1005735)
Supplement: S8 Table — (PDF) [file pgen.1005735.s014.pdf]

Supplementary Table S8: Oligonucleotides

Strain construction

NPL3 URA3 integration

NPL3-URA3-fwd

AAAAAGGACATGAGAAAAATAATTTCCTCTCTTCTAAATATATATACTTTTGAAGGAATCAAAATTAA
GGATTGTACTGAGAGTGCACCA

NPL3-URA3-rev

GAGCCTGTGGTGGTTCTTGGTGCTGTTCTTCGACTGGGGCATCGACAACAGATTCTGGTAGTTGCTCT
ACTGTGCGGTATTTCACACCGC

PTH URA3 replacement

NPL3(promoter)-PTH-fwd

GGATTTAAAAAGGACATGAGAAAAATAATTTCCTCTCTTCTAAATATATATACTTTTGAAGGAATCAA
AATTAAGCAATTACGCTAAAACCATAAGGATAATGAACAAATTCAACAAAGA

NPL3-rev

GCCATGAACAACCTTCTGGT

NPL3 deletion

NPL3-KO-fwd

AAGGAATCAAAATTAAGCAATTACGCTAAAACCATAAGGACGGATCCCCGGGTTAATTAA

Npl3-KO-rev

TGTTTTCCTTTTTCATTTGTTCTCAGTCTCATATTTAAGGAATTCGAGCTCGTTTAAAC

Oligonucleotides for CRAC

5' linkers (3 random nucleotides, plus barcodes), IDT:

L5Ad	5'-invddT-ACACrGrArCrGrCrUrCrUrUrCrCrGrArUrCrUrNrNrNrCrGrCrUrUrArGrC-OH-3'
L5Bb	5'-invddT-ACACrGrArCrGrCrUrCrUrUrCrCrGrArUrCrUrNrNrNrGrUrGrArGrC-OH-3'
L5Bc	5'-invddT-ACACrGrArCrGrCrUrCrUrUrCrCrGrArUrCrUrNrNrNrCrArCrUrArGrC-OH-3'
L5Bd	5'-invddT-ACACrGrArCrGrCrUrCrUrUrCrCrGrArUrCrUrNrNrNrUrCrUrCrUrArGrC-OH-3'
L5Ca	5'-invddT-ACACrGrArCrGrCrUrCrUrUrCrCrGrArUrCrUrNrNrNrCrUrArGrC-OH-3'
L5Cb	5'-invddT-ACACrGrArCrGrCrUrCrUrUrCrCrGrArUrCrUrNrNrNUrGrGrArGrC-OH-3'
L5Cc	5'-invddT-ACACrGrArCrGrCrUrCrUrUrCrCrGrArUrCrUrNrNrNrArCrUrCrArGrC-OH-3'
L5Cd	5'-invddT-ACACrGrArCrGrCrUrCrUrUrCrCrGrArUrCrUrNrNrNrGrArCrUrUrArGrC-OH-3'

3' linker (pre-activated, adenylated linker for cloning), IDT:

miRCat-33™ 3'-L

rAppTGGAATTCTCGGGTGCCAAGG/ddC/

RT and PCR primers

miRCat-33™ RT

CCTTGGCACCCGAGAATT

PCR fwd

AATGATACGGCGACCACCGAGATCTACACTCTTTCCCTACACGACGCTCTTCCGATCT

PCR rev

CAAGCAGAAGACGGCATACGAGATCGGTCTCGGCATTCCTGGCCTTGGCACCCGAGAATTCC

Strand-specific RT-QPCRs

Reverse transcription primers

VHR2_RT	CTGAAGAACTGGGCCTTGTC
THO1	GAGCGGTTGTTTGCATTTTT
UPF2_RT	TTTAAACACCCCCAGAGACG
PTC7	ACCACCGCTGTAGTTTTTGC
CYC1	CCTTCTTCAACCCACCAAAG
SNR3_RT	TTGAGACGCAGCTGAAAAGA
EFM3	TCGTTGTTTGCGTCGTACTC

QPCR primers

VHR2 fwd	AATGAGGCCACACTCCACAT
VHR2 rev	CTATCTGCCGATGTGCTTGA
THO1 fwd	GAAACAAACGAACCCAAGGA
THO1 rev	GATCCAATGCTTTGGCCTTA
UPF2 fwd	GGTGATCTTGACGCAGACAG
UPF2 rev	CTTCCGCTTTCCTTTCTTCC
PTC7 fwd	ACGCTTTTAACGCCAAAGAA
PTC7 rev	GATGGGAAGTGAGCCACAAT
CYC1 fwd	CTCTGGTCAAGCTGAAGGGT
CYC1 rev	TTCTTCAACCCACCAAAGGC
SNR3 fwd	CCACCGCATTAGACAGTACG
SNR3 rev	ACCCGGAAATTGATCTCTCC
EFM3 fwd	AGACCACGAATGGATTTTCG
EFM3 rev	CGTCTTTCATCATCGGGTCT
ACT1 fwd	GTTACGTCGCCTTGGACTTC
ACT1 rev	TGGGGCTCTGAATCTTTCGT

Northern probe

SNR60	GCGAAAGACTAATTTCGATGG
